# Intestinal mucosal alterations parallel central demyelination and remyelination: insights into the gut-brain axis in the cuprizone model of multiple sclerosis

**DOI:** 10.3389/fimmu.2025.1682183

**Published:** 2025-10-28

**Authors:** Carolina Ferreira, Filipa Carvalho, Pedro Vieira, André Alves, Filipe Palavra, Jani Almeida, Vera Alves, Ezequiel Coscueta, Patrícia Dias Pereira, Manuela Pintado, Helena Sá, Miguel Castelo-Branco, Flávio Reis, Sofia Viana

**Affiliations:** ^1^ Institute of Pharmacology and Experimental Therapeutics, Faculty of Medicine, University of Coimbra, Coimbra, Portugal; ^2^ Coimbra Institute for Clinical and Biomedical Research (iCBR), Faculty of Medicine, University of Coimbra, Coimbra, Portugal; ^3^ Center for Innovative Biomedicine and Biotechnology (CIBB), University of Coimbra, Coimbra, Portugal; ^4^ Clinical Academic Center of Coimbra (CACC), Coimbra, Portugal; ^5^ Polytechnic Institute of Coimbra, ESTESC-Coimbra Health School, Pharmacy, Coimbra, Portugal; ^6^ Centre for Child Development - Neuropediatrics Unit, Hospital Pediátrico, Unidade Local de Saúde de Coimbra, Coimbra, Portugal; ^7^ Institute of Immunology, Faculty of Medicine (FMUC), University of Coimbra, Coimbra, Portugal; ^8^ Universidade Católica Portuguesa, CBQF - Centro de Biotecnologia e Química Fina – Laboratório Associado, Escola Superior de Biotecnologia, Porto, Portugal; ^9^ Instituto de Ciências Biomédicas Abel Salazar da Universidade do Porto (ICBAS-UP), School of Medicine and Biomedical Sciences, University of Porto, Porto, Portugal; ^10^ LAQV-REQUIMTE, Associated Laboratory for Green Chemistry, University of Porto, Porto, Portugal; ^11^ Nephrology Department, Centro Hospitalar e Universitário de Coimbra, Unidade Local de Saúde (ULS) Coimbra, Coimbra, Portugal; ^12^ Coimbra Institute for Biomedical Imaging and Translational Research (CIBIT)-Institute for Nuclear Sciences Applied to Health (ICNAS), University of Coimbra, Coimbra, Portugal; ^13^ Institute of Physiology, Faculty of Medicine, University of Coimbra, Coimbra, Portugal

**Keywords:** cuprizone-induced demyelination, remyelination, gut-brain axis, gut microbiota, intestinal innate and adaptative immunity, multiple sclerosis, neuroinflammation, gliosis

## Abstract

**Background:**

The gut-brain axis has been increasingly recognized as a critical factor in Multiple Sclerosis (MS) pathophysiology. While its role in demyelination is well documented, gut-brain axis involvement during remyelination remains largely unexplored.

**Methods:**

Using the cuprizone (CPZ) model, which induces reversible demyelination and spontaneous remyelination upon toxin withdrawal, we investigated gut and brain changes during both disease stages in C57BL/6 mice. Animals were administered 0.2% cuprizone for 5 weeks to induce demyelination, followed by a 2-week recovery phase. Intestinal changes were evaluated through 1) gut microbiota profiling and metabolite production (short-chain fatty acids (SCFAs), indoxyl sulfate), 2) structural and barrier integrity via histology, mucus staining, and tight junction markers (ZO-1, occludin, claudin-5), 3) mucosal immunity through M1/M2 macrophage profiling and Th17/Treg ratios, and 4) expression of inflammatory and oxidative stress markers. Differences in brain demyelination/remyelination, gliosis and related molecular changes were determined using immunohistochemistry and real-time polymerase chain reaction (RT-PCR).

**Results:**

The demyelination peak was characterized by reduced abundance of SCFA-producing genus *Akkermansia* and *Dubosiella*, increased intestinal permeability at the level of the mucus layer and tight junction networks, and shifts in mucosal immunity toward a pro-inflammatory state characterized by M1 macrophages and Th17 cell expansion together with elevated levels of inflammatory cytokines (IL-17, IL-1β) and changes in oxidative stress-related enzymes (iNOS, HO-1, SOD1/2), all of which were partially reversed during the remyelination phase. Centrally, cuprizone-induced demyelination/remyelination and gliosis showed region-specific patterns. Neuroinflammation peaked during demyelination (TNF-α, IL-1β, IL-6, IL-17) and only partially resolved, suggesting that a balanced inflammatory response may aid remyelination.

**Conclusion:**

Our findings reveal that cuprizone-induced intestinal dysfunctions temporally parallel central nervous system (CNS) lesion dynamics, disclosing temporal coordination of both compartments and highlighting gut-brain axis impact on both disease stages.

## 1 Introduction

Multiple Sclerosis (MS) is a chronic immune-mediated disorder of the central nervous system (CNS) and spinal cord, marked by focal demyelination, neuroinflammation, and progressive axonal degeneration. The disease is characterized by alternating phases of relapse and remission, marked by cycles of demyelination and spontaneous, but often incomplete, remyelination. However, as the disease progresses, remyelination becomes increasingly ineffective. Importantly, current therapies primarily target inflammation and immune modulation, but do not directly address remyelination failure, which remains a major therapeutic challenge.

Beyond its well-known neurological manifestations, a significant proportion of MS patients experience gastrointestinal (GI) symptoms, particularly fluctuation between constipation and fecal incontinence, suggesting broader systemic involvement (1–3). Histological intestinal abnormalities, namely villous atrophy, immune infiltration, and smooth muscle hypertrophy have been observed in MS patients (4, 5). These changes mirrored what is observed in the experimental autoimmune encephalomyelitis (EAE) model, where intestinal barrier disruption closely correlates with disease severity (6, 7). In this context, the gut-brain axis has emerged as a pivotal player in MS pathophysiology (8–10), offering novel insights into comprehension of disease mechanisms and opening new avenues for therapeutic intervention.

The gut-brain axis encompasses the bidirectional communication network established between the GI tract and the CNS, mediated through hormonal, neural, metabolic, and immunological pathways (11). Mounting evidence from both clinical and preclinical studies has highlighted the influence of gut microbiota (GM) composition and function on CNS autoimmunity (12, 13). Over the past decade, the GM has emerged as a critical modulator of immune homeostasis and neuroinflammatory processes, with increasing evidence implicating its dysregulation - dysbiosis - in the pathogenesis of MS. MS patients often present altered gut microbial compositions characterized by reduced abundance of beneficial commensals (e.g., *Faecalibacterium*, *Parabacteroides*, *Bifidobacterium*, *Prevotella*) and an increase in pro-inflammatory taxa such as *Ruminococcus* and *Methanobrevibacter* (14, 15), which correlate with a pro-inflammatory T cell profile that may exacerbate autoimmune responses (12). These microbial shifts are thought to influence peripheral immunity through modulation of intestinal barrier function and microbial metabolite production, particularly SCFAs and tryptophan metabolites. These immune modulator compounds are known to promote regulatory T cell differentiation, reinforce epithelial integrity, and dampen inflammatory pathways, all of which may play protective roles in MS (16–20).

A growing body of evidence from both clinical and preclinical studies has firmly established a role for the gut–brain axis in the demyelination process of MS (21–24). In animal models, particularly EAE, modulation of the GM through germ-free conditions, antibiotic treatment, or dietary interventions has been shown to influence peripheral immune priming and disease severity, including the extent and distribution of CNS demyelination (25–27).

These findings support a functional gut–immune–brain communication axis, whereby intestinal microbial and immune alterations may directly modulate neuroinflammatory responses. While most studies have focused on the contribution of the gut to demyelination, emerging albeit less abundant evidence suggests that the gut–brain axis may also play a role in the remyelination process. Indeed, a few recent studies have proposed that specific microbial signatures, microbial metabolites such as SCFAs or tryptophan derivatives, and gut-derived immune signals may influence oligodendrocyte precursor cell (OPC) differentiation and remyelination efficiency (28–33). Accordingly, there is a pressing need for preclinical models to enable systematic, phase-specific investigation of the gut–brain axis across both demyelination and remyelination stages. The CPZ intoxication model represents a valuable complementary approach to autoimmune models such as EAE. CPZ administration in rodents induces a highly reproducible, non-immune-mediated pattern of demyelination, predominantly affecting the corpus callosum and hippocampus, that is followed by spontaneous remyelination upon toxin withdrawal (34). Its primary mechanism of action involves mitochondrial dysfunction and oxidative stress, leading to selective oligodendrocyte apoptosis (34). Notably, CPZ is administered orally and exhibits minimal systemic absorption (35), suggesting that the gastrointestinal tract is directly and chronically exposed to this compound. Despite this, the extent to which CPZ-induced demyelination affects intestinal mucosal homeostasis, including barrier function and immune signaling, remains poorly understood. While a few studies have documented gut microbiota alterations in CPZ-treated animals (24, 36), comprehensive assessment of the intestinal mucosa, particularly across the full demyelination-remyelination cycle, has not yet been undertaken.

Building on this rationale, the present study aimed to perform a temporal and integrative characterization of the gut–brain axis components in the CPZ model of demyelination followed by spontaneous remyelination. By evaluating GM composition and function, epithelial barrier integrity, mucosal immune activation, and inflammatory signaling in parallel with CNS demyelination and remyelination dynamics, we seek to uncover potential temporal patterns between intestinal alterations and central nervous pathology.

## 2 Materials and methods

### 2.1 Animals and treatments

24 C57BL/6J male mice (8 weeks old) were acquired from Charles River Laboratories, Barcelona, Spain. Animals were housed in the animal facility of Coimbra Institute for Clinical and Biomedical Research (iCBR), Faculty of Medicine, University of Coimbra, four per cage, under controlled environmental conditions with day/night cycles of 12 h, temperature 22±1°C and relative humidity 50-60% with food and tap water supplied *ad libitum*. After 2 weeks of acclimatization, acute demyelination was induced by daily administering 0.2% (m/v) CPZ (bis-cyclohexanone oxaldihydrazone; Sigma-Aldrich, C9012) dissolved in methylcellulose 1% (w/v) by oral gavage, for 5 weeks (W5, n=8). Cuprizone was freshly prepared each day over the 5 weeks of the experiment. To foment early remyelination, cuprizone administration was stopped and mice allowed to recover for 2 more weeks (W7, n=8). Vehicle only was administered to control animals (n=8). All animals were monitored weekly for body weight (BW), food and water consumption. Animal experiments were conducted according to the National and European Communities Council Directives of Animal Care, received approval (#12/2018) by the local Animal Welfare Body (ORBEA) and were reported according to ARRIVE guidelines (37).

### 2.2 Sample collection and tissue preparation

At the end of weeks 5 and 7, animals were sacrificed via intraperitoneal injection with 10:1 mixture of ketamine (100 mg/mL; Ketaset, Fort Dodge, Fort Dodge, IA) and xylazine (100 mg/mL; Anased, Lloyd Laboratories, Shenandoah, IA), and immediately transcardially perfused with cold phosphate-buffered saline (PBS). Whole brains, guts, thymuses, and livers were carefully extracted and weighed (**Supplementary Table 1**). Each brain was bisected into two hemispheres: the left hemispheres of all animals were immersed in RNA later (Sigma-Aldrich, R0901) for gene expression analysis (n=8); right hemispheres were cryoprotected in 30% sucrose prepared in PBS for 3 days and incorporated in OCT CryoMatrix (ThermoScientific, 6769006) for fluorescence microscopy (n=4).

Peyer’s patches were excised from the intestinal surface for multiparametric flow cytometry analysis (n=8). Prior to dissection, the intestines were mechanically cleared of luminal contents. Fecal samples were collected and stored for 16S rRNA sequencing and SCFA quantification. Isolated Peyer’s patches were thoroughly rinsed with PBS to remove mucus, mechanically disrupted, and passed through a 70-µm nylon cell strainer (Corning, 352235) using gentle pressure to obtain single-cell suspensions.

Following Peyer’s Patches’ excision, both the small intestine and colon were divided into designated segments. Colon sections were processed through the gut bundling technique, as previously described (38) and embedded in paraffin for widefield microscopy or OCT CryoMatrix for immunohistochemistry analysis (n=4).

All samples were preserved at -80°C until further utilization.

### 2.3 Gene expression analysis

Total RNA was extracted from 30–50 mg of frozen brain left hemispheres and colonic tissue using the NZY Total RNA Isolation Kit (NZYTech, MB13402), following the manufacturer’s instructions. Complementary DNA (cDNA) was synthesized using the Xpert cDNA Synthesis Mastermix (GRISP, GK81.0100, Lot. 7E2709A).

RT-PCR was performed with the CFX96 Touch Real-Time PCR Detection System (Bio-Rad, 1845096). For brain samples, specific forward and reverse primers were used to amplify myelin basic protein (MBP) and proteolipid protein (PLP). In intestinal samples, primers targeted occludin, zonula occludens-1 (ZO-1), claudin 5, nuclear factor kappa B p65 subunit (NF-κB p65), NF-κB repressing factor (NKRF), and transforming growth factor beta 1 (TGF-β).

Additionally, for both brain and intestinal tissues, primers were used for tumor necrosis factor alpha (TNF-α), interleukins 1 beta (IL-1β), 6 (IL-6), and 17 (IL-17), inducible nitric oxide synthase (iNOS), heme oxygenase-1 (HO-1), and superoxide dismutase 1 (SOD1) and 2 (SOD2).

Each RT-PCR reaction consisted of 10 μL of SYBR Green Supermix (Bio-Rad, iTaq Universal SYBR Green Supermix, 1725124), 7.6 μL of distilled water, 0.4 μL of primer mix, and 2 μL of cDNA. The thermocycling protocol was the following: initial denaturation at 95°C for 10 minutes, followed by 40 cycles of 15 seconds at 95°C, 45 seconds at either 58°C or 60°C (depending on primer specificity), and 30 seconds at 72°C.

Gene expression was normalized using the GeNorm algorithm, with hypoxanthine phosphoribosyltransferase (HPRT) and glyceraldehyde 3-phosphate dehydrogenase (GAPDH) serving as reference genes. Relative expression levels were calculated using the 2^–ΔΔCt method and expressed as a percentage of the control group. Primer sequences are listed in **Table 1**.

**Table 1:** Primer sequences and real-time PCR conditions.

Primer sequence			
Gene	Forward	Reverse	Temperature (°C)
GAPDH	CGA CTT CAA CAG CAA CTC	TGT AGC CGT ATT CAT TGT CAT A	58
HPRT	TCC ATT CCT ATG ACT GTA	CAT CTC CAC CAA TAA CTT	58
MBP	GCC TGT CCC TCA GCA GAT TT	GTC GTA GGC CCC CTT GAA TC	58
PLP	CAG GCA GAT CTT TGG CGA CT	GTG ATG CCC ACA AAC GTT GC	60
Occludin	TTG AAC TGT GGA TTG GCA GC	AAG ATA AGC GAA CCT TGG CG	60
Claudin-5	TTA AGG CAC GGG TAG CAC TC	TAC TTC TGT GAC ACC GGC AC	60
ZO-1	GGA TTT ACC CGT CAG CCC TT	ATG CTG GGC CTA AGT ATC CC	60
NF-kB p65	AGGCTTCTGGGCCTTATGTG	TGCTTCTCTCGCCAGGAATAC	58
Nkrf	AGACCAGCCTGTAGCAACCAACAT	GCTTCTCGGCAACCAAGGACTCA	60
TGF-β1	CCGCAACAACGCCATCTAT	CCAAGGTAACGCCAGGAATT	60
TNF-α	ATG GCC TCC CTC TCA TCA GT	TGG TTT GCT ACG ACG TGG G	60
IL-1β	GGA CAG ATT ATC AAC CAA CA	ACA CAG GAC AGG TAT AGA TT	60
IL-6	CTC TGC AAG AGA CTT CCA TCC A	TGG AAA TTG GGG TAG GAA GGA C	60
IL-17	GGA CTC TCC ACC GCA AAT GAA	TTT CCC TCC GCA TTG ACA CA	60
iNOS	CAC CTT GGA GTT CAC CCA GT	ACC ACT CGT ACT TGG GAT GC	58
HO-1	GAC AGA AGA GGC TAA GAC CGC	TGA CGA AGT GAC GCC ATC T	58
SOD1	AAC CAG TTG TGT TGT CAG GAC	CCA CCA TGT TTC TTA GAC TGA GG	60
SOD2	CAG ACC TGC CTT ACG ACT ATG G	CTC GGT GGC GTT GAG ATT GTT	60

Forward and reverse primer sequences used for quantitative real-time PCR (qRT-PCR), along with annealing temperatures for each target gene. All primers were designed for mouse genes and validated for specificity.

### 2.4 DNA extraction from fecal samples

Microbial DNA was extracted from approximately 200 mg of fecal samples (n=4) using the NZY Tissue gDNA Isolation Kit (NZYtech, MB13502), following the manufacturer’s protocol with minor modifications. Briefly, samples were pre-treated with TE buffer and lysozyme (10 mg/mL) and incubated at 37°C for 2 hours to enhance lysis of bacterial cell walls. After centrifugation, the pellet was treated with Proteinase K and incubated at 56°C for 2 hours. DNA binding was performed using NL buffer and ethanol, followed by washing steps with NW2 buffer to remove contaminants. DNA was eluted in 100 µL of preheated NE buffer and quantified. Extracted DNA was used for downstream Illumina sequencing. Quality control was performed at each step to ensure data reliability.

### 2.5 16S rRNA sequencing and bioinformatic analysis

The V3–V4 region of bacterial 16S rRNA was amplified using primers CCTAYGGGRBGCASCAG (forward) and GGACTACNNGGGTATCTAAT (reverse). Amplicons were verified through 2% agarose gel electrophoresis, pooled equimolarly, and prepared for sequencing following end-repair, A-tailing, and adapter ligation. Library quality and fragment size were checked using a Qubit Fluorometer and Agilent 2100 Bioanalyzer. Sequencing was performed on an Illumina NovaSeq platform to generate paired-end 250 bp reads.

Raw reads were processed using the QIIME2 pipeline (version 2022.2). Quality filtering, denoising, merging, and chimera removal were performed with DADA2, producing Amplicon Sequence Variants (ASVs). Taxonomic assignment was based on the SILVA 138 database. Sequencing depth was sufficient for all samples, with a normalization cutoff of 22,950 reads per sample. Rarefaction curves were plotted for each sample and indicated adequate coverage, leveling off at approximately 2,500 reads. Alpha diversity and beta diversity (Bray–Curtis distances, PCoA) were calculated to assess microbial richness and community structure and phylogenetic trees were built from multiple sequence alignments. Functional predictions were inferred using PICRUSt2.

Statistical analyses were conducted using QIIME2 and GraphPad Prism. Differences in alpha diversity were assessed using the Kruskal–Wallis test or Mann–Whitney U test and beta diversity differences were evaluated via PERMANOVA. Taxonomic differences at various levels (phylum, genus, species) were tested using ANCOM or LEfSe, with *p*-values adjusted for multiple comparisons (Benjamini–Hochberg FDR correction). A *p*-value< 0.05 was considered statistically significant.

### 2.6 SCFAs quantification

#### 2.6.1 Standard and calibration curve preparation

Stock solutions of SCFAs were prepared in diethyl ether at concentrations of 7.00 x 10³ μM for acetic, propionic, and butyric acids, and 1.00 x 10³ μM for *i*-butyric, valeric and *i*-valeric acids. An internal standard stock solution was prepared by dissolving 24 μL of isocaproic acid (iC6) in 10 mL of diethyl ether to yield a final concentration of 19.8 x 10³ μM. Calibration curves were generated using a 2-fold serial dilution series prepared from the stock solutions. Each standard was spiked with internal standard to a final concentration of 330 μM. All solutions were stored at 4°C until analysis.

#### 2.6.2 SCFAs extraction from fecal samples

Fecal samples (n=4) were collected immediately after deposition, frozen at -80°C, and stored until use. For extraction, samples were thawed, homogenized, and 100 mg of each sample was acidified with 0.25 mL of 50% (w/v) sulfuric acid and vortexed for 3 minutes. A 50 μL aliquot of internal standard solution (19.8 x 10³ μM) was added to each sample to reach a final concentration of 330 μM. SCFAs were extracted by adding 1 mL of diethyl ether, followed by centrifugation at 2800 × *g* for 5 minutes. The extraction was repeated twice, and the organic phases were combined in a total volume of 3 mL.

#### 2.6.3 Gas chromatography analysis

SCFA concentrations were determined using the gas chromatograph Agilent 8860 (Agilent, USA) equipped with a flame ionization detector and a BPX70 capillary column (60 m x 0.25 mm x 0.25 μm; SGE Europe Ltd, Courtaboeuf, France). Compound identification was based on retention times and mass spectra comparison with authentic standards.

### 2.7 Measurement of urinary indican

Indican (indoxyl sulfate, I3S) levels in urine samples (n=4) were determined using the Indican Assay Kit (MAK128, Sigma-Aldrich), following the manufacturer’s instructions.

Briefly, 50 μL of each urine sample was transferred to individual wells of a clear 96-well plate, followed by the addition of 140 μL of Reagent A. The plate was gently mixed, and absorbance was measured at 480 nm (A480) to obtain the blank reading.

Next, 10 μL of Reagent B was added to each well, mixed, and incubated for 5 minutes at room temperature before measuring absorbance again to obtain the sample reading. To generate the standard curve, 10 μL of the provided standard solution (30 mg/dL) was added to separate wells and processed identically.

Indican concentration was calculated using the formula provided by the manufacturer:


$$\text{Indican}\;\left(\text{mg}/\text{dL}\right)=\frac{\left(\text{A}480_{sample}-\text{A}480_{blank}\right)\;\times 5\;\times \text{ n}}{\text{A}480_{standard}-\text{A}480_{sample}}$$

where *n* represents the sample dilution factor. If calculated values exceeded 20 mg/dL, samples were diluted in water and reanalyzed, with the final concentration adjusted for the dilution factor.

All assays were performed in duplicate, and absorbance was read using a spectrophotometric microplate reader set at 480 nm. Quality control measures were applied throughout to ensure result accuracy.

### 2.8 Colon histological analysis

#### 2.8.1 Hematoxylin and eosin staining

Tissue samples (n = 4) were fixed in formalin and embedded in paraffin wax. A single HE-stained cryosection (5 µm thick) from each block was analyzed. Sections were deparaffinized with xylene and rehydrated through a descending ethanol gradient to distilled water. Subsequently, the slides were stained with Gill’s hematoxylin solution No. 1 (Sigma Aldrich) for 2 minutes, rinsed in tap water, and counterstained with 0.5% aqueous eosin (Sigma Aldrich) for 30 seconds. After staining, the sections were dehydrated, cleared, and coverslipped. All samples were evaluated under a Zeiss microscope Mod. Axioplan 332 2 (Zeiss, Jena, Germany) and photographed in three to five representative regions at x5, x10 and x40 magnifications. The obtained images were used to measure the thickness of the mucosa, submucosa, muscularis and the whole intestinal wall. All the measures were carried out by the same person, using the free ImageJ^®^ software version 1.54f (https://imagej.net/). In each sample, the layer thickness was measured at five points, and the average value was used for analysis. Changes in mucosal architecture, including the presence of inflammatory infiltrates, were also evaluated.

#### 2.8.2 Alcian Blue-Periodic Acid Schiff staining

Colon tissue samples (n=4) were sectioned at 5 μm thickness and mounted onto positively charged slides. Sections were rehydrated sequentially using xylene (ThermoFisher Scientific) three times, followed by 100% FLEX twice, 95% FLEX, 70% FLEX, and 50% FLEX, each for 3 minutes.

Mucin staining was performed to differentiate neutral and acidic mucins. Alcian Blue (0.05%, ThermoFisher Scientific) prepared in 3% acetic acid was applied for 15 minutes, followed by rinsing under running tap water for 3 minutes and then distilled water for 2 minutes. Sections were then treated with 0.05% periodic acid for 5 minutes, rinsed under running tap water for 3 minutes, and further washed in distilled water for 5 minutes.

Subsequently, slides were stained with Schiff’s reagent (Ricca Chemical Company, Arlington, TX, USA) for 10 minutes, rinsed under running tap water for 3 minutes, and distilled water for 5 minutes. Finally, Nuclear Fast Red was applied for 5 minutes to stain cell nuclei, followed by washing under running tap water for 3 minutes and distilled water for 5 minutes. Slides were air-dried and coverslipped using Permount^®^ mounting medium (ThermoFisher Scientific).

All samples were examined by light microscopy using a Zeiss microscope Mod. Axioplan 332 2 (Zeiss, Jena, Germany). Four to six micrographs per slide were captured at x40 magnification using identical manual microscope settings and white balance for all images to ensure consistency. Three representative images per animal were selected for subsequent quantitative analysis.

Color intensity quantification of the colon was performed using TissueQuant software (39). Prior to analysis, images were cropped in Adobe Photoshop to isolate individual crypt. From each image, four crypts were selected from left to right, outlined, and saved as separate files for TissueQuant analysis. Within TissueQuant, specific parameters were set to detect and quantify the colors deep blue, dark blue/purple, and magenta. For each crypt, color density was calculated as the sum of the products of color value and area for the three colors. Goblet cell numbers were manually counted per crypt. Mucus density was determined by dividing the total color sum by the crypt area. Mucin content per goblet cell was calculated by dividing total mucin density by the number of goblet cells. Goblet cell density was expressed as the number of goblet cells per unit crypt area.

### 2.9 Immunohistochemical analysis of intestinal barrier integrity

Colon samples (n=4) were fixed overnight in 4% paraformaldehyde, embedded in OCT CryoMatrix and sectioned at 10 µm. Cryosections of OCT-embedded intestines were fixed in cold acetone (-20°C) for 10 minutes, followed by three washes in PBS for 5 minutes each to remove residual fixative. Sections were then permeabilized with 0.25% Triton X-100 in PBS for 15 minutes and washed again three times in PBS. To block non-specific binding sites, sections were incubated with a blocking solution containing 10% fetal bovine serum (FBS) and 0.25% Triton X-100 in PBS for 40 minutes at room temperature, followed by additional PBS washes. Sections were then incubated overnight at 4°C with primary antibodies mouse anti-occludin (1:100; Invitrogen, 33-1500) and rabbit anti-ZO-1 (1:100; Invitrogen, 40-2200) diluted in 3% FBS in PBS. After washing three times in PBS, sections were incubated with secondary antibodies goat anti-mouse 488 (1:1000; Invitrogen, A11029) and goat anti-rabbit 594 (1:1000; Invitrogen, A11037) for 1 hour at RT. Secondary antibodies were prepared in PBS with DAPI (1:10000, Invitrogen, D1306). The slides were washed again and mounted using Glicergel mounting medium for imaging.

All images were acquired with a Zeiss microscope Mod. Axioplan 332 2 (Zeiss, Jena, Germany) at a magnification of x20. Two colonic sections per animal stained for occludin and ZO-1 were imaged, and ten images per animal were acquired. Non-overlapping fields were randomly selected from colonic crypts with preserved tissue integrity. Folded and artefact-containing regions were excluded. To minimize quantification bias, we focused on colonic crypts of similar size across samples and blind scoring was performed.

Staining intensity was measured using the software ImageJ version 1.54f. The photomicrographs were transformed to grayscale, and a threshold was set to obtain binary images. Afterwards, relative staining intensities of occludin and ZO-1 were semi-quantified in binary converted images, and the results were transformed into percentage area values.

### 2.10 Macrophage immunolabeling and quantification

For macrophage identification, colonic sections (n=4) were incubated with the following primary antibodies, diluted in 3% FBS in PBS: rabbit anti-CD68 (1:500; Abcam, ab125212) to label total macrophages, mouse anti-CD86 (1:100; Abcam, ab220188) for M1 macrophages, and goat anti-CD206 (1:100; Bio-Techne, AF2535) for M2 macrophages. After three PBS washes, sections were incubated for 1 hour at RT with species-specific secondary antibodies: donkey anti-rabbit Alexa Fluor 594 (1:1000; Invitrogen, R37119), donkey anti-mouse Alexa Fluor 488 (1:1000; Invitrogen, A21202), and donkey anti-goat Alexa Fluor 647 (1:1000; Invitrogen, A21447), prepared in PBS containing DAPI (1:10,000; Invitrogen, D1306) for nuclear staining.

### 2.11 Multiparametric flow cytometry

Images were captured using a Zeiss Axioplan 332–2 microscope (Zeiss, Jena, Germany) at x20 magnification. For each animal, two colonic sections per animal stained for CD68, CD86 and CD206 were imaged (x20 magnification) and five non-overlapping fields per section were randomly selected from colonic crypt areas with preserved tissue integrity. Folded and artefact-containing regions were excluded. To minimize quantification bias, we focused on colonic crypts of similar size across samples and blind scoring was performed. Positive cells for CD68, CD86 and CD206 were manually counted using ImageJ Cell Counter plugin, following the method described by Chiloeches et al. (40). The numbers of M1 and M2 macrophages were expressed as a percentage of total CD68+ macrophages.

Peyer’s patches were excised from mechanically cleaned intestines (n=8), washed in PBS to remove mucus, mechanically disrupted in PBS, and filtered through a 70-μm nylon cell strainer (Corning, Cat. No. 352235) to obtain single-cell suspensions. Cells were counted using a DxH 500 hematology analyzer (Beckman Coulter) and adjusted to 10×10^6^ cells/mL in 1× PBS.

For multiparametric staining, 1×10^6^ cells were incubated with the following extracellular fluorochrome-conjugated monoclonal antibodies (mAbs) for 20 minutes at room temperature (RT) in the dark: anti-CD3-FITC, anti-CD4-PE-Cy7, anti-CD25-PE, and anti-CD45-APC-Cy7. Cells were then washed with BD Pharmingen™ Stain Buffer (554656) and centrifuged at 250×g for 10 minutes.

Fixation was carried out using BD Pharmingen™ Fixation Buffer (Mouse Foxp3 Buffer Set, 560409) for 30 minutes at 4°C in the dark, followed by centrifugation at 500×g for 5 minutes. Cells were subsequently washed with pre-warmed BD Pharmingen™ Permeabilization Buffer, centrifuged, and incubated in the same buffer for 30 minutes at 37°C in the dark for permeabilization. After washing, intracellular staining was performed using anti-FoxP3-AF647, anti-RORγt-BV421, and anti-IL-17-BV510 for 20 minutes at RT in the dark.

Following final washes, data acquisition was performed on a BD FACSCanto™ II flow cytometer using BD FACSDiva™ software, collecting at least 15,000–25,000 CD4^+^ T cells per sample. For viability assessment, 0.25×10^6^ cells were stained with BD Pharmingen™ 7-Amino-Actinomycin D (7-AAD, 559925) for 30 minutes at 4°C in the dark and acquired separately. Detailed information on the antibodies used is provided in **Supplementary Table 2**.

Data were analyzed using FlowJo^®^ v10.7 (BD Biosciences). Doublets were excluded via FSC-H/FSC-A, lymphocytes gated as CD45^+^/SSC-A low, and CD3^+^CD4^+^ T cells identified. Regulatory T cells (Tregs, CD45^+^CD3^+^CD4^+^CD25^+^FoxP3^+^) and Th17 cells (CD45^+^CD3^+^CD4^+^IL-17^+^RORγt^+^) were defined as previously described (40). The gating strategy is shown in **Supplementary Figure 1**.

### 2.12 Immunohistochemical analysis of brain myelination and gliosis

To measure cuprizone-induced demyelination and glial responses, brain serial 30 μm sagittal sections were cut on a cryostat (n=3). The sections were stored as free floating at -20°C in cryopreservation solution, consisting of 30% glycerol and 30% ethylene glycol in phosphate buffer 0.4M. To label myelin, microglia and astrocytes, sections were fixed with 4% paraformaldehyde, washed 3 times for 5 minutes in PBS and then blocked and permeabilized in 5% FBS and 0.25% Triton X-100 in PBS at RT for 2 hours. Slices were then incubated overnight at 4°C on a shaker in primary antibody prepared in 1% FBS. The primary antibodies used were rabbit anti-MBP (1:250, Abcam, ab40390) for myelin, rabbit anti-IBA-1 (1:200, Wako chemicals, 019–19741) for microglia, and mouse anti GFAP (1:250; MERCK, IF03L-100UG) for astrocytes. Microglia and astrocytes were co-stained in the same sections. After washing 3 times for 10 mins in PBS, slices were incubated at RT on a shaker for 2 hours in secondary antibodies: goat anti-rabbit Alexa Fluor 594 (1:1000, Invitrogen, A11037) for MBP, goat anti-rabbit Alexa Fluor 488 (1:1000, Invitrogen, A11034) for Iba-1 and donkey anti-mouse Alexa Fluor 594 (1:1000, Thermo Fisher, A21203) for GFAP. Secondary antibodies were prepared in PBS with DAPI (1:10000, Invitrogen, D1306). Slices were then washed 3 times in PBS and mounted on glass slides using Dako S3023 Fluorescence Mounting Medium.

All images were acquired with a Zeiss microscope Mod. Axioplan 332 2 (Zeiss, Jena, Germany) at x20 magnification. Four sagittal brain sections per animal stained for GFAP/IBA-1 and one to two sections stained for MBP were imaged for each region of interest: corpus callosum, hippocampus and cortex.

For staining intensity measurement, a semi-automated densitometrical analysis was performed with the software ImageJ version 1.54f. The photomicrographs were transformed to grayscale, and a threshold was set to obtain binary images. Afterwards, relative staining intensities of MBP, GFAP and IBA-1 were semi-quantified in binary converted images, and the results were transformed into percentage area values.

### 2.13 Fractal analysis of glial cells

In order to evaluate glial morphology, 4 sagittal brain sections stained for IBA-1 and GFAP from each animal were imaged for the three regions of interest: corpus callosum, hippocampal region CA1 and the cortical layer 5/6. A Zeiss microscope Mod. Axioplan 332 2 (Zeiss, Jena, Germany) was used to acquire all the three-dimensional fluorescent images. The images were acquired as stacks of ~40 slices with at 0.75 μm z-stack step size intervals using a x40 (NA 1.3) oil immersion objective, which were later combined to obtain a single high-quality image displaying detailed magnification of the cell processes. Images were typically 1024 by 1024 pixels and covered a square field of view 212.55 to 212.55 μm wide.

Microglia and astrocytes fractal analysis was performed as described (41). Briefly, each image was processed in a systematic way to obtain a filled image and its counterpart outlined shape. For this purpose, a series of steps were performed using ImageJ free software 1.54f and appropriate plugins (**Supplementary Figure 2**): colorized images (**Supplementary Figure 2a**) were transformed to 8-bit grayscale to best visualize all positive staining (**Supplementary Figure 2b**); were filtered to soften the background (**Supplementary Figure 2c**) and enhance the contrast (**Supplementary Figure 2d**); despeckeled to remove salt-and-pepper noise generated in the previous steps (**Supplementary Figure 2e**); binarized to obtain a black and white image by applying an established threshold (**Supplementary Figure 2f**), despeckeled once again (**Supplementary Figure 2g**); subjected to the close function to connect dark pixels separated by up to 2 pixels (**Supplementary Figure 2h**), and targeted for outliers (**Supplementary Figure 2i**). Taking the images from each animal, a total of 6 cells per brain area were randomly selected and cropped, in conformity with the following criteria: (1) no overlapping with neighboring cells and (2) completely visible nucleus and branches. Selection was done blinded to treatment. Then, with the aim of obtaining a cell image formed by a single and continuous set of pixels, each image was manually edited: some pixels were cleared to separate ramifications pertaining to neighboring cells, while others were added to join processes belonging to the selected cell (compare **Supplementary Figure 2j, k**). The binary image was then processed to extract and save the outlined shape for further analysis (**Supplementary Figure 2l**).

In order to quantify the morphological changes of microglia cells and astrocytes during the course of CPZ intoxication, four parameters were measured resorting to the plugin Fraclac for ImageJ (available at imagej.net/ij/plugins/fraclac/fraclac.html), through box-counting scan: (1) fractal dimension, (2) lacunarity, (3) density, and (4) convex hull area.

### 2.14 Statistical analysis

Data were expressed as means±standard deviation(SDand analyzed using GraphPad Prism^®^ version 9.5.0 (730) (GraphPad Software, La Jolla, CA, USA). The Shapiro-Wilk test was applied to assess the normality of data distribution. For normally distributed data, one-way ANOVA followed by Bonferroni’s *post hoc* test was used for multiple group comparisons. For non-normally distributed data, the Kruskal-Wallis test with Dunn’s multiple comparisons test was applied. Repeated measures ANOVA, followed by Bonferroni’s *post hoc* test, was used to assess changes over time. All pairwise comparisons were performed. A p-value< 0.05 was considered statistically significant.

## 3 Results

### 3.1 Phase-specific gut microbial signatures during demyelination and remyelination

Aiming to explore the intestinal component of the CPZ model, GM composition was assessed through 16S rRNA sequencing of fecal samples.

**Figure 1:**
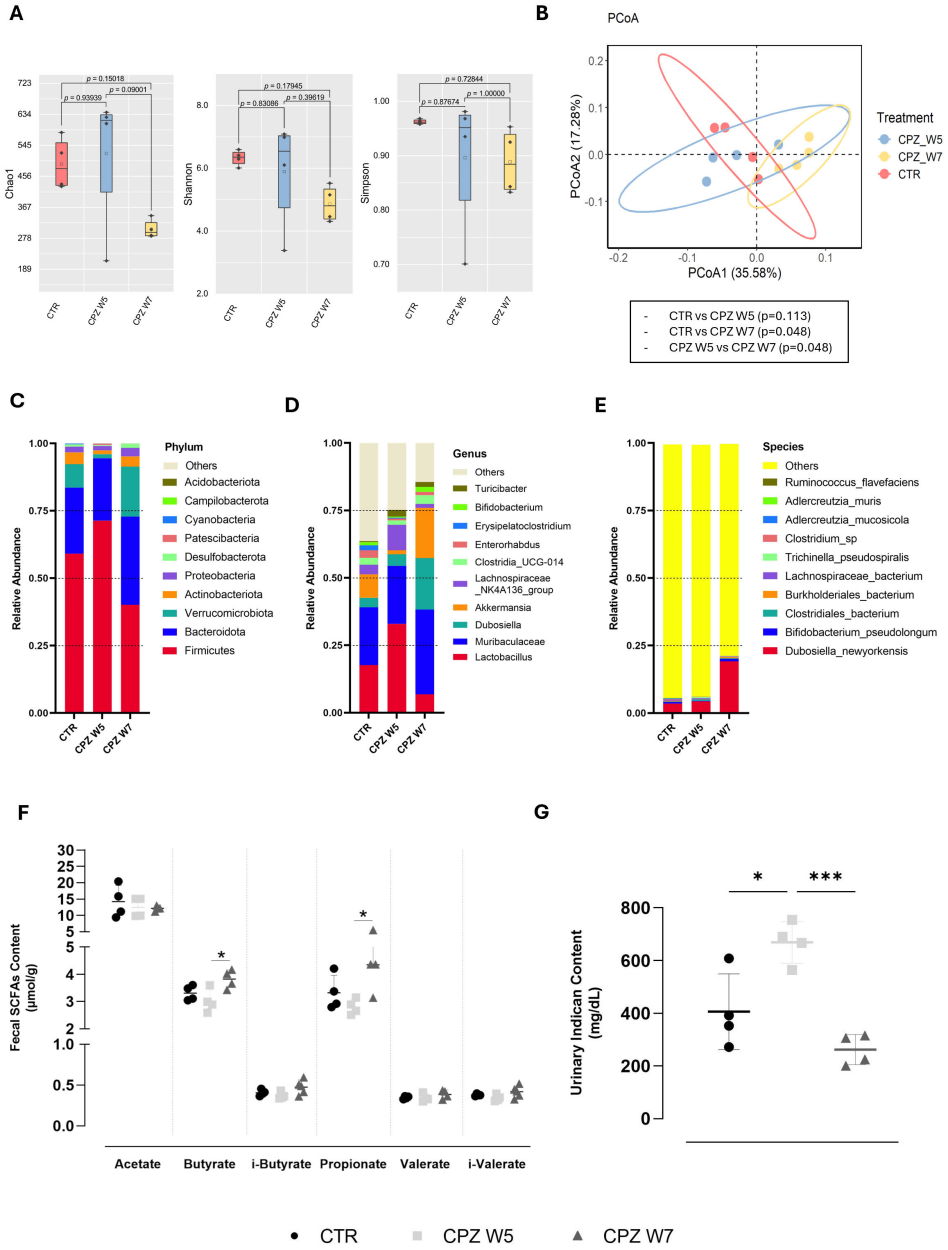
Gut microbiota diversity and taxonomic composition in cuprizone-intoxicated mice. **(A)** Alpha diversity indices (Chao1, Shannon, and Simpson) reflecting species richness and evenness in control mice (CTR), cuprizone-treated mice at the demyelination peak (CPZ W5), and during the remyelination phase (CPZ W7). **(B)** Beta diversity assessed by Principal Coordinates Analysis (PCoA) based on weighted UniFrac distances, illustrating differences in microbial community structure between groups. **(C–E)** Relative abundance of the ten most abundant taxa at the phylum **(C)**, genus **(D)**, and species **(E)** levels. **(F)** Concentration of short-chain fatty acids (SCFAs) in fecal content. **(G)** Concentration of indoxyl sulfate (indican) in urine. Data is presented as mean ± S.D. of 4 animals per group. Statistical comparisons were performed between all groups. Symbols are absent for non-significant comparisons. *p < 0.05, ***p < 0.005.

Although cuprizone administration did not result in statistically significant shifts in gut microbial composition, distinct trends emerged. Alpha diversity indices - Chao1, Shannon, and Simpson (**Figure 1A**) – revealed that while microbial richness and diversity were not significantly impacted in the demyelination peak (Week 5), a compositional shift was detected during the remyelination phase (Week 7). Nonetheless, beta diversity analysis (**Figure 1B**) showed a partial separation between control and demyelinated animals, suggesting that toxin exposure might influence the intestinal microbial community. By the remyelination phase, despite remaining separate from the controls, the microbial landscape appeared to shift closer to baseline, implying some degree of compositional recovery.

At the taxonomic level, demyelination was associated with an increased abundance of the phylum *Firmicutes* and reductions in *Verrucomicrobiota* and *Actinobacteria* (**Figure 1C**), which appeared to revert upon toxin withdrawal. These phylum-level changes were mirrored at the genus level, with elevated levels of *Lactobacillus*, *Muribaculaceae*, and *Lachnospiraceae NK4A136*, and a relative decline in *Akkermansia* during demyelination (**Figure 1D**). In contrast, the remyelination phase was characterized by higher abundances of *Dubosiella* and *Akkermansia*. Specifically, *Dubosiella newyorkensis* exhibited a marked increase during this phase (**Figure 1E**).

To determine whether these microbial shifts were functionally relevant, we assessed the levels of gut-derived bacterial metabolites, including fecal SCFAs (**Figure 1F**) and the uremic toxin indoxyl sulfate (indican), a tryptophan-derived product, in urine (**Figure 1G**). While SCFA levels showed a modest, non-significant decrease during the demyelination, they rebounded in the remyelination phase, significantly so for butyrate (p = 0.0300) and propionate (p = 0.0256). In parallel, urinary indican levels increased during demyelination (p = 0.0145), and returned to baseline upon remyelination (p = 0.0008).

### 3.2 Dynamic remodeling of goblet cell colonic mucus accompanies demyelination and remyelination

**Figure 2:**
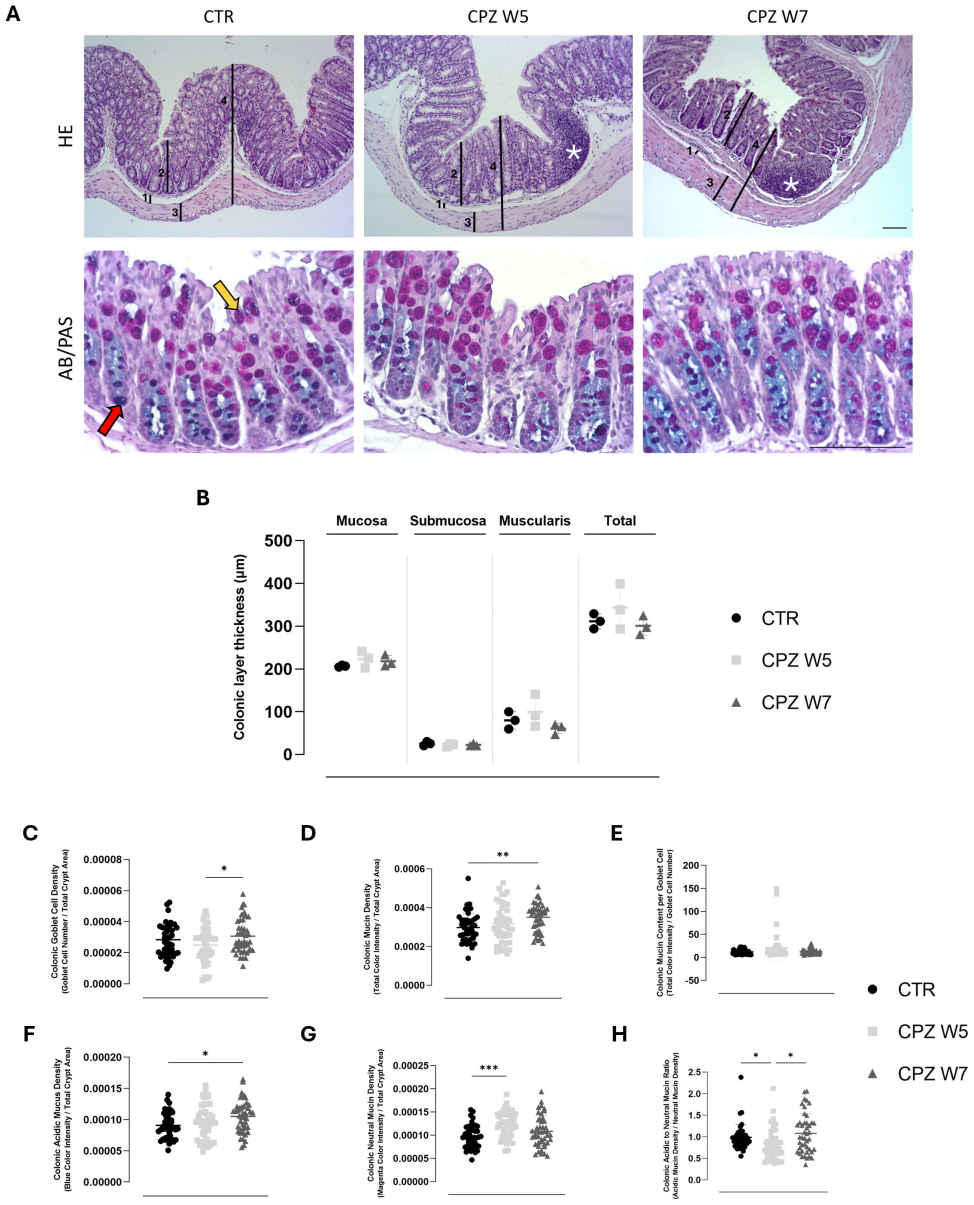
Histological assessment of colonic architecture through histomorphometric analysis of colonic wall layers, inflammatory cell infiltration, and mucin composition in cuprizone-intoxicated mice. **(A)** Representative hematoxylin and eosin (HE)-stained colonic sections from controls (CTR), CPZ-intoxicated mice at the demyelination peak (CPZ W5), and CPZ-intoxicated mice during the remyelination phase (CPZ W7), depicting the distinct histological layers: 1 – Submucosa, 2 – Mucosa, 3 – Muscularis, 4 – Total intestinal wall, used for thickness measurements. Cellular infiltrates indicative of inflammation are marked by asterisks (top);Representative Alcian Blue-Periodic Acid Schiff (AB/PAS)-stained colonic sections from the same experimental groups, showing mucin-producing goblet cells (bottom). The red arrow indicates acidic mucins (blue staining), while the yellow arrow indicates neutral mucins (magenta staining). Scale bar = 100 μm. **(B)** Histomorphometric measurements of submucosa, mucosa, muscularis, and total colonic wall thickness, determined from H&E-stained sections. Data are presented as mean±S.D. of 3 animals per group. Statistical comparisons were performed between all groups. Summary of mucus-related parameters, including **(C)** goblet cell density, **(D)** mucin density, **(E)** mucin content per goblet cell, **(F)** acidic mucin density, **(G)** neutral mucin density and **(H)** acidic to neutral mucin ratio, assessed from Alcian Blue/Periodic Acid-Schiff (AB/PAS)-stained sections using TissueQuant. Data is presented as mean±S.D. of 4 animals per group. Statistical comparisons were performed between all groups. Symbols are absent for non-significant comparisons. *p< 0.05; **p< 0.01; ***p< 0.005.

HE-stained colonic sections were analyzed to investigate potential structural alterations induced by CPZ exposure (**Figure 2**– upper panel). No significant differences in the thickness of the colonic layers, including mucosa, submucosa, muscularis, and total colonic wall, were detected among the experimental groups, indicating preserved tissue architecture throughout the demyelination and remyelination phases. Nonetheless, histological examination revealed increased number of lymphoid aggregates in the colonic mucosa of CPZ-intoxicated animals, a feature not found in control mice, suggesting local immune reactivity despite the lack of other morphological changes.

In parallel, the intestinal mucus - recognized as a critical component of gut barrier integrity - was evaluated using Alcian Blue/PAS staining (**Figure 2** – bottom panel) and morphometric quantitative analysis was conducted using TissueQuant and ImageJ.

Following CPZ administration, a modest but non-significant reduction in goblet cell number was observed, which normalized after toxin withdrawal (**Figure 2C**). Interestingly, this phase was marked by a trend toward increased total mucin density (**Figure 2D**) and elevated mucin content per goblet cell (**Figure 1E**), particularly for neutral mucins (p = 0.0002) (**Figure 2G**). During the remyelination phase, mucin content per cell returned to baseline, yet a concurrent rise in both goblet cell number (p = 0.0457) and mucin density (p = 0.0095) was detected. Notably, this increase was driven predominantly by acidic mucins (p = 0.0229) (**Figure 2F**). These dynamics were captured by a significant drop in the acidic-to-neutral mucin ratio during demyelination (p = 0.0462), which normalized upon remyelination, underscoring transient vulnerability of the mucus phenotype (p = 0.0202) (**Figure 2H**).

### 3.3 Synchronized regulation of intestinal junctional integrity during demyelination and remyelination

**Figure 3:**
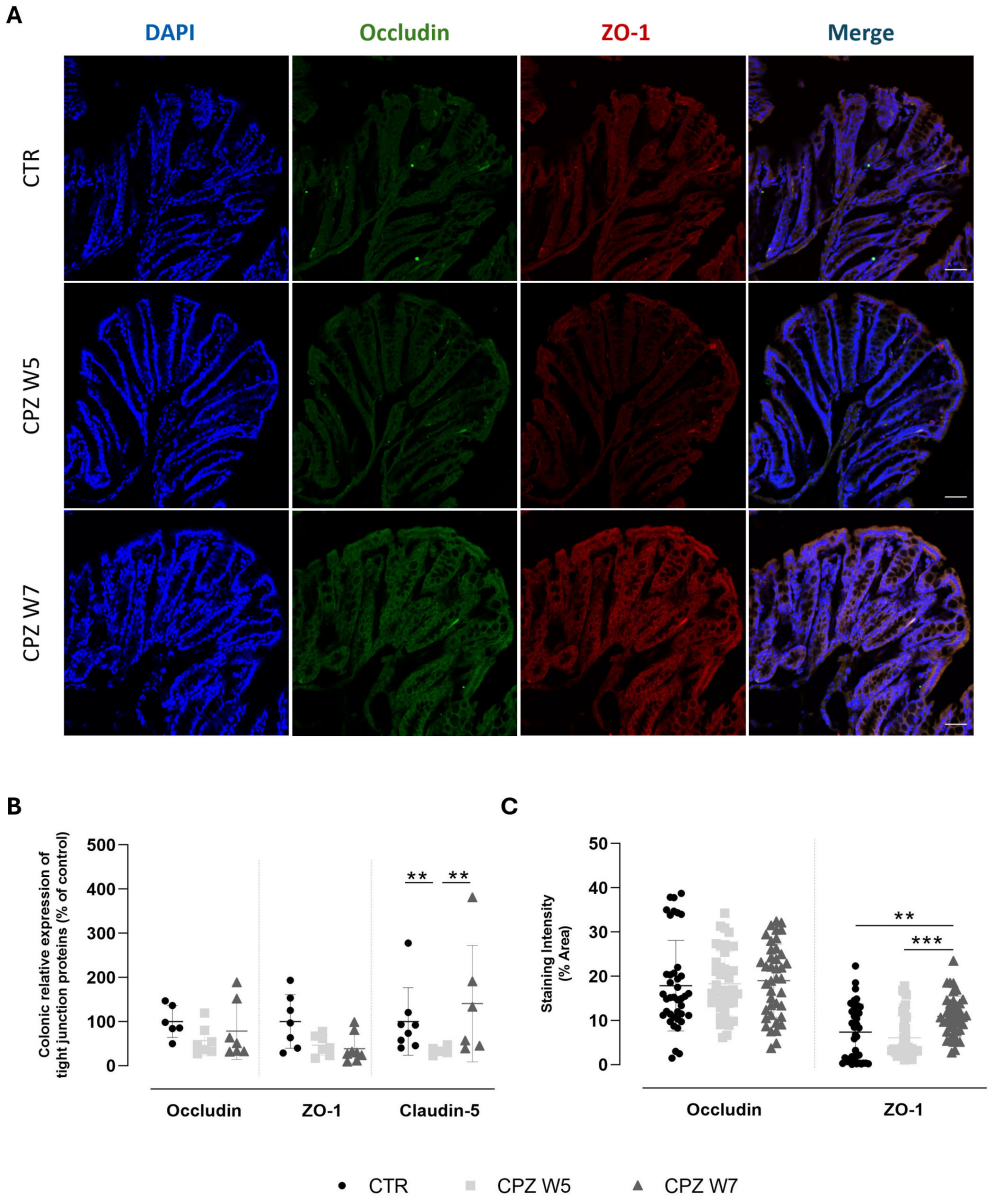
Expression and localization of tight junction proteins in the colon of cuprizone-intoxicated mice. **(A)** Representative photomicrographs of DAPI (blue), occludin (green), ZO-1 (red) and immunostaining of controls (CTR), CPZ-intoxicated mice at the demyelination peak (CPZ W5), and CPZ-intoxicated mice at the remyelination phase (CPZ W7). Scale bar = 50 µm. **(B)** mRNA levels of tight junction proteins in the colon. **(C)** Quantification of occludin- and ZO-1-positive staining areas in the colon. Data is presented as mean±S.D. of 4 animals per group. Statistical comparisons were performed between all groups. Symbols are absent for non-significant comparisons. **p< 0.01, ***p< 0.005.

Tight junctions are essential for maintaining epithelial cohesion and regulating paracellular permeability. To further examine the integrity of the gut barrier, the gene and protein expression of key tight junction components - occludin, claudin-5, and ZO-1 - were analyzed. Representative images are shown in **Figure 3A**. RT-PCR data revealed a general downregulation of tight junction gene expression in response to CPZ, with partial recovery following toxin cessation (**Figure 3B**). Claudin-5 was the only marker to show a statistically significant decline upon demyelination (p = 0.0340) as well as restoration during the remyelination phase (p = 0.0496). At the protein level (**Figure 3C**), immunohistochemical analysis showed stable expression of occludin throughout CPZ exposure, whereas ZO-1 was significantly upregulated during remyelination (W7) (p = 0.0057 versus CTR; p = 0.0006 versus CPZ W5) (**Figure 3C**), suggesting possible compensatory remodeling or post-transcriptional regulation mechanisms that stabilize the barrier during recovery. These findings point to a complex and layered regulation of junctional integrity in response to cuprizone-induced stress.

### 3.4 Temporal coordination of innate and adaptive immune compartments in gut-associated lymphoid tissue over the course of demyelination and remyelination

**Figure 4:**
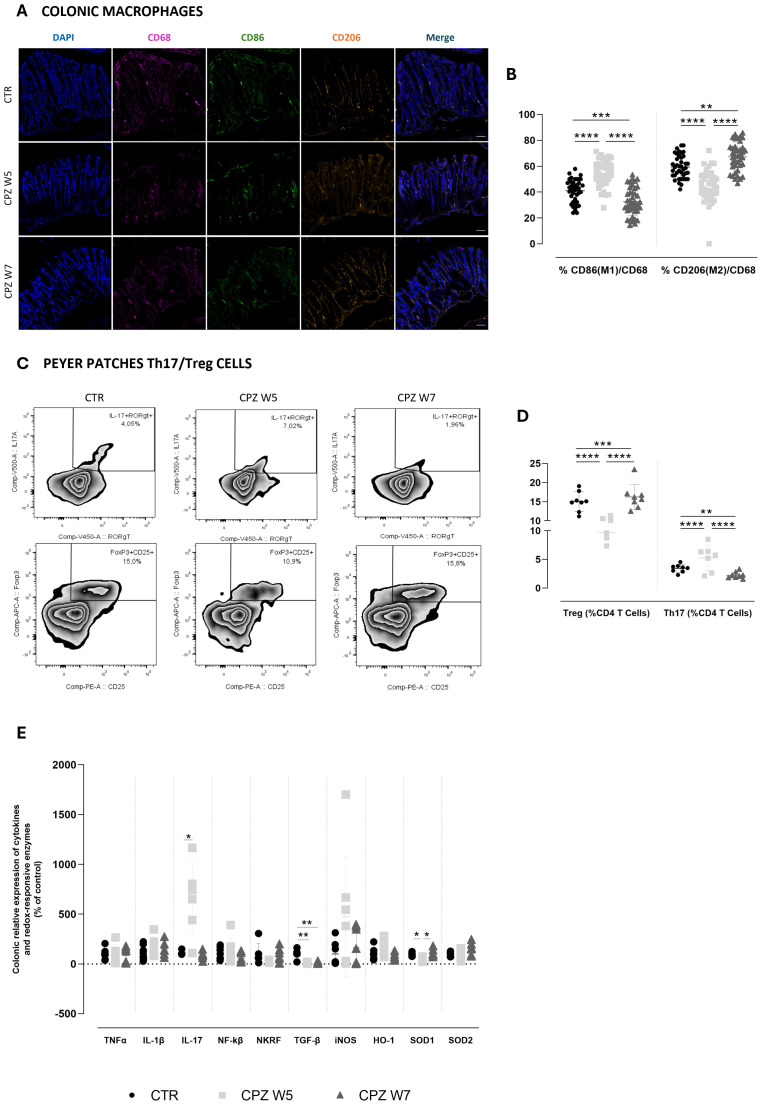
Gut immune, inflammatory and oxidative dynamics in cuprizone-intoxicated mice. **(A)** Representative photomicrographs of DAPI (blue), CD68 (pink), CD86 (green), and CD206 (orange) triple immunostaining of controls (CTR), CPZ-intoxicated mice at the demyelination peak (CPZ W5), and CPZ-intoxicated mice at the remyelination phase (CPZ W7) in the colon. Scale bar = 50 μm. **(B)** Summary of the proportion of M1 (CD86^+^) and M2 (CD206^+^) macrophages, expressed as a percentage of total CD68^+^ macrophages. Data is presented as mean±S.D. of 4 animals per group. **(C)** Representative flow cytometry zebra plots of IL-17^+^ RORγt ^+^ (upper) and Foxp3^+^ CD25 ^+^ cells (lower) gated on CD3^+^CD4^+^ T cells from small intestinal Peyer patches suspensions. **(D)** Summary data and statistical analysis of the relative frequency of IL-17^+^ RORγt ^+^ and Foxp3^+^ CD25 ^+^ cells within CD3^+^CD4^+^ T cells. Data is presented as mean±S.D of 8 animals per group. (**(E)** mRNA expression levels of pro-inflammatory cytokines (TNF-α, IL-1β, IL-17, NF-κB, NKRF and TGF-β) and redox-responsive enzymes (iNOS, HO-1, SOD1, and SOD2) in colonic tissue. Data is presented as mean±S.D of 6–8 animals per group. Statistical comparisons were performed between all groups. Symbols are absent for non-significant comparisons. *p<0.05; **p<0.01; ***p<0.005; ****p<0.001.

Given the observed alterations in GM composition, microbial metabolites, and mucus dynamics, we next investigated whether these changes were accompanied by shifts in intestinal immune compartments, focusing on colonic macrophage polarization and T cell populations within gut-associated lymphoid tissue (GALT) (**Figure 4**).

In the colonic mucosa, macrophage polarization was assessed via immunohistochemistry using CD68 (total macrophages), CD86 (M1, pro-inflammatory phenotype), and CD206 (M2, anti-inflammatory phenotype) markers (**Figure 4A**). Quantitative analysis revealed that during the demyelination peak (week 5), the proportion of CD86^+^ M1 macrophages significantly increased (p< 0.0001), whereas CD206^+^ M2 macrophages were markedly reduced compared to controls (p< 0.0001), indicating a shift toward a pro-inflammatory macrophage profile. This polarization pattern was reversed in the remyelination phase (week 7), as M1 macrophage levels declined below those of control animals (p< 0.0001 versus CPZ W5; p = 0.0008 versus CTR) while M2 macrophage proportions significantly rose (p< 0.0001 versus CPZ W5; p = 0.0019 versus CTR), suggestive of an anti-inflammatory, tissue-repairing milieu (**Figure 4B**). Total macrophage counts are summarized in **Supplementary Figure 3**.

Parallel assessment of adaptive immunity in Peyer’s patches by flow cytometry further reflected this dynamic immune modulation (**Figure 4C**). During demyelination, a substantial rise in IL-17^+^ RORγt^+^ CD4^+^ Th17 cells (p = 0.0249) was accompanied by a decrease in Foxp3^+^ CD25^+^ CD4^+^ Tregs (p = 0.0052) (**Figure 4D**), indicating a pro-inflammatory skewing of the intestinal T cell balance. Upon remyelination, this imbalance was normalized, restoring the Th17/Treg ratio toward homeostatic levels (p = 0.0006 and p = 0.0007, respectively). Together, these results demonstrate that CPZ intoxication induces enhanced intestinal pro-inflammatory polarization during demyelination and a compensatory resolution phase during remyelination.

Finally, we characterized the colonic inflammatory and redox status by assessing the relative gene expression of key cytokines and redox-responsive elements (**Figure 4E**). Analysis of these mediators revealed that, at the demyelination peak (W5), the colon exhibited a predominantly pro-inflammatory gene expression profile. This was characterized by an upregulation of IL-17A, a trend toward increased IL-1β, and reduced expression of TGF-β (p = 0.0076), a pivotal regulator of intestinal immune tolerance. In parallel, animals showed a trend toward elevated NF-κB expression and decreased levels of its repressor Nkrf. In the remyelination phase (W7), most inflammatory markers demonstrated a reversal of these patterns, consistent with partial resolution of inflammation and reflecting the dynamic nature of cuprizone-associated myelin damage and repair. However, this resolution appeared incomplete, as TGF-β expression remained suppressed (p = 0.0073 versus CTR). Notably, TNF-α expression remained relatively unchanged throughout the experiment, indicating it may not play a central role in the intestinal response to CPZ.

In parallel with the inflammatory changes, oxidative stress-related gene expression was also assessed to further understand the intestinal response to CPZ intoxication. Both iNOS and HO-1 exhibited increased expression levels at the demyelination peak, which then declined toward baseline during remyelination, although these changes did not reach statistical significance. This pattern suggests an early activation of antioxidant and nitric oxide-related pathways in response to cuprizone-induced intestinal stress. Conversely, SOD1 expression was significantly downregulated during demyelination (p = 0.0423), indicating a compromised enzymatic antioxidant defense at this stage. Notably, SOD1 levels fully recovered during remyelination (p = 0.0105), paralleling the resolution of oxidative stress. A milder, non-significant increase was also observed for SOD2 during the recovery phase, further supporting partial restoration of mitochondrial antioxidant capacity.

### 3.5 Region-specific spatiotemporal patterns of demyelination and remyelination

**Figure 5:**
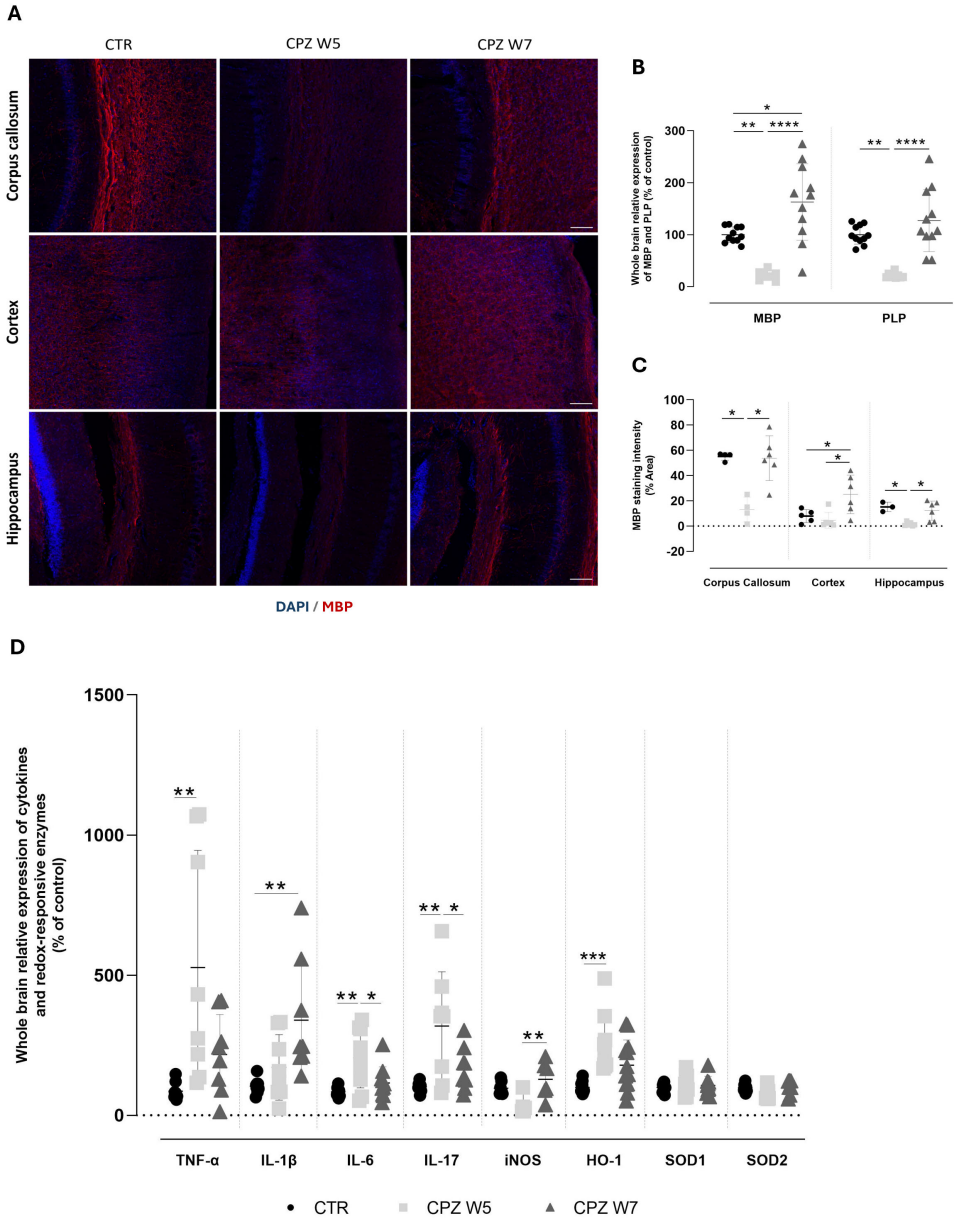
Myelination status and neuroinflammatory profile in the brain of cuprizone-intoxicated mice. **(A)** Representative photomicrographs of MBP (red) and DAPI (blue) double immunostaining in the corpus callosum (top), cortex (middle), and hippocampal CA1 region (bottom) of control mice (CTR), cuprizone-treated mice at the demyelination peak (CPZ W5), and during the remyelination phase (CPZ W7). Scale bar: 100 μm. **(B)** mRNA expression levels of myelin-related genes (MBP and PLP) in whole-brain tissue. Data is presented as mean±S.D of 3 animals per group. **(C)** Quantification of MBP-positive area. **(D)** Expression levels of pro-inflammatory cytokines (TNF-α, IL-1β, IL-6, IL-17) and redox-responsive enzymes (iNOS, HO-1, SOD1, SOD2) in whole-brain tissue. Data are presented as mean±S.D of 6–8 animals per group. Statistical comparisons were performed between all groups. Symbols are absent for non-significant comparisons. *p< 0.05, **p< 0.01, ***p< 0.005, ****p< 0.001.

At the CNS, demyelination was assessed by immunolabelling 30 μm sagittal brain cryosections for MBP (**Figure 5A**) as well as by analyzing gene expression levels of myelin basic protein (MBP) and proteolipid protein (PLP) (**Figure 5B**) in whole brain. Signal intensity was quantified in the corpus callosum, the somatosensory cortex, and the CA1 region of the hippocampus (**Figure 5C**).

As expected, after 5 weeks of cuprizone administration, mice exhibited a significant reduction in the expression levels of both *Mbp* (p = 0.0032) and *Plp* genes (p = 0.0022). Following the 2-week recovery period without CPZ, the expression of both genes returned to baseline levels (p< 0.0001), indicating full recovery. MBP protein levels mirrored the gene expression results, with cuprizone inducing a significant reduction in MBP immunoreactivity across all analyzed brain regions, significantly so in the corpus callosum (p = 0.0347) and the hippocampus (p = 0.0165) However, this decrease was markedly less pronounced in the cortex. Additionally, while cuprizone withdrawal led to near-complete remyelination in the corpus callosum (p = 0.0387) and hippocampus (p = 0.0347), MBP levels in the cortex were found to be even higher after the 2-week recovery period than before cuprizone exposure (p = 0.0221 versus CTR, p = 0.0283 versus CPZ W5).

To further characterize the neuroinflammatory response during demyelination and remyelination, we assessed the expression of key brain inflammatory and oxidative stress-related markers (**Figure 5D**). At the peak of demyelination, there was a significant upregulation of pro-inflammatory cytokines, including TNF-α (p = 0.0090), IL-6 (p = 0.0075), and IL-17 (p = 0.0017), with IL-1β showing a similar trend that did not reach statistical significance (p = 0.2011). Interestingly, during the remyelination phase, IL-1β expression increased significantly (p = 0.0022), while TNF-α (p = 0.4644), IL-6 (p = 0.0289), and IL-17 (p = 0.0191) levels showed partial recovery toward baseline. Concurrently, oxidative stress-related enzymes exhibited distinct expression patterns. Mice exposed to cuprizone displayed a marked upregulation of HO-1 (p = 0.0008) that was partially reversed following toxin withdrawal, suggesting a dynamic regulation in response to redox imbalance. In contrast, iNOS expression was reduced during demyelination (p = 0.1301) but significantly elevated during the remyelination phase (p = 0.0142), while SOD expression remained largely unchanged throughout the course of cuprizone exposure.

### 3.6 Cuprizone-induced demyelination triggers lasting morphological changes in astrocytes

**Figure 6:**
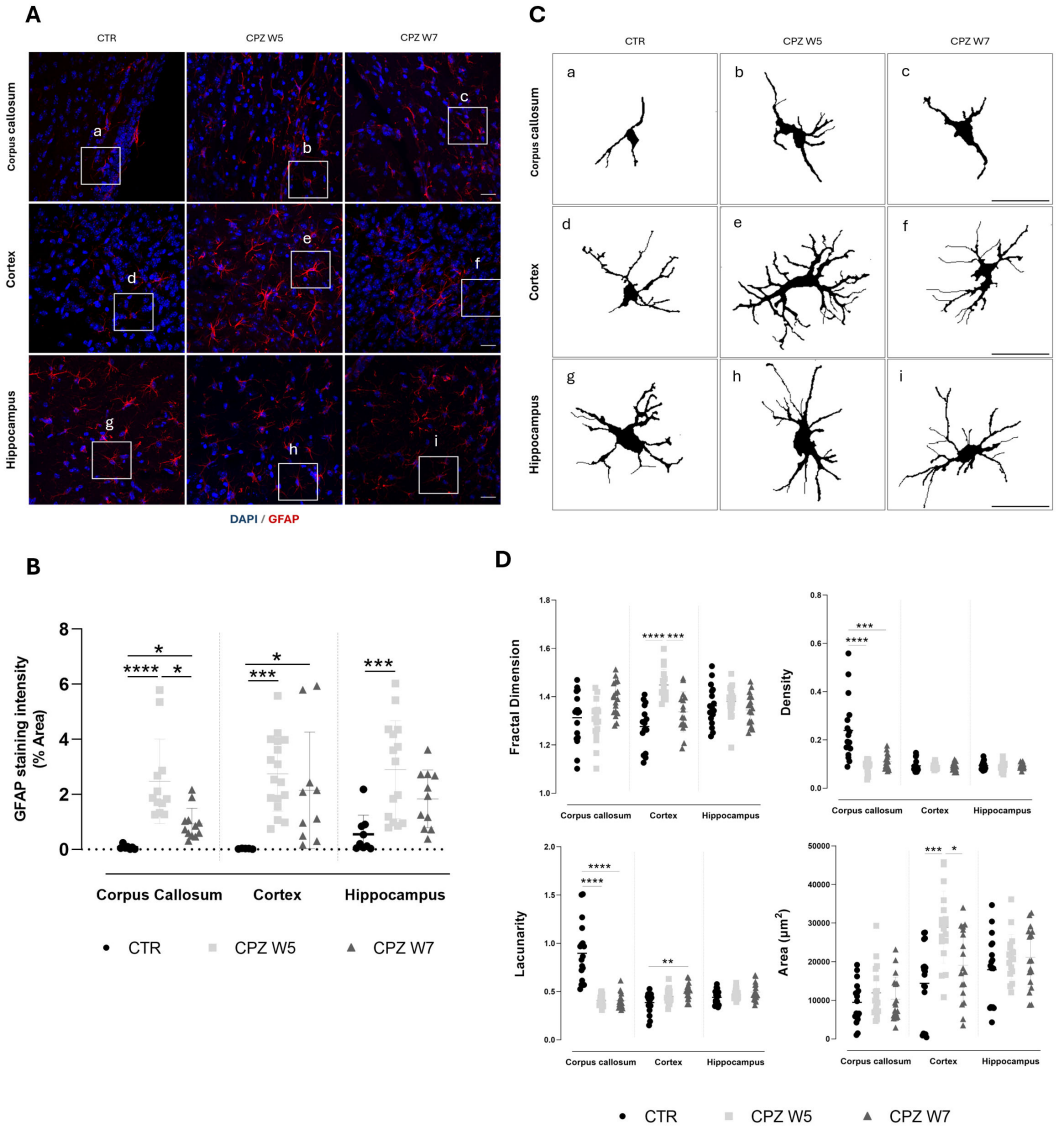
Astrogliosis in the brain of cuprizone-intoxicated mice. **(A)** Representative z-stacked photomicrographs of DAPI (blue) and GFAP (red) staining in astrocytes from control (CTR) mice, CPZ-intoxicated mice at the demyelination peak (CPZ W5), and CPZ-intoxicated mice during the remyelination phase (CPZ W7) in the corpus callosum (upper panel), cortex layer VI (middle panel), and hippocampal CA1 region (lower panel). Scale bar: 20 μm. **(B)** Quantification of GFAP-positive area. The region marked by a square in **(A)** is shown at higher magnification in **(C)**, where individual astrocyte outlines were extracted for fractal analysis to assess morphological complexity. **(D)** Summary data and statistical analysis of astrocyte fractal dimension, lacunarity, density, and area. Data is presented as mean±S.D. of 3 animals per group. Statistical comparisons were performed between all groups. Symbols are absent for non-significant comparisons. *p< 0.05, **p< 0.01, ***p< 0.005, ****p< 0.001.

To assess whether regional myelin remodeling parallels astrocytic remodeling, brain cryosections were immunolabeled for GFAP (**Figure 6A**). Quantitative analyses, including signal intensity and fractal dimension measurements, were performed in the corpus callosum, somatosensory cortex, and the CA1 region of the hippocampus.

A robust and sustained upregulation of GFAP expression in astrocytes was observed at the peak of demyelination across all brain regions (**Figure 6B**). Although attenuated during the remyelination phase, astrogliosis persisted, particularly in the cortex. To further investigate how astrocytes’ activation, defined by changes in cellular morphology, is affected during CPZ-induced demyelination and remyelination, fractal analysis was conducted using Fiji (**Figure 6C, D**). At the peak of demyelination, the somatosensory cortex exhibited significant increases in fractal dimension (p< 0.0001) and cell area (p = 0.0005), both of which partially normalized during remyelination (p = 0.0007 and p = 0.0301, respectively), suggesting a transient activation of cortical astrocytes in response to CPZ, followed by a return to baseline upon resolution of damage. Interestingly, astrocytes in the corpus callosum appeared denser and more heterogeneous than those in the cortex and hippocampus at baseline. Their fine, extended processes are arranged in parallel tracts, contributing to increased density and lacunarity (**Figure 6C**) due to their alignment with axons and vasculature. In response to cuprizone intoxication, both astrocyte density and lacunarity in the corpus callosum decreased significantly (p< 0.0001), consistent with astrocytic hypertrophy (**Figure 6D**). In contrast to other brain regions, hippocampal astrocytes exhibited no significant morphological changes throughout the course of CPZ intoxication.

### 3.7 Differential microglial responses reflect regional vulnerability to cuprizone-induced CNS injury

**Figure 7:**
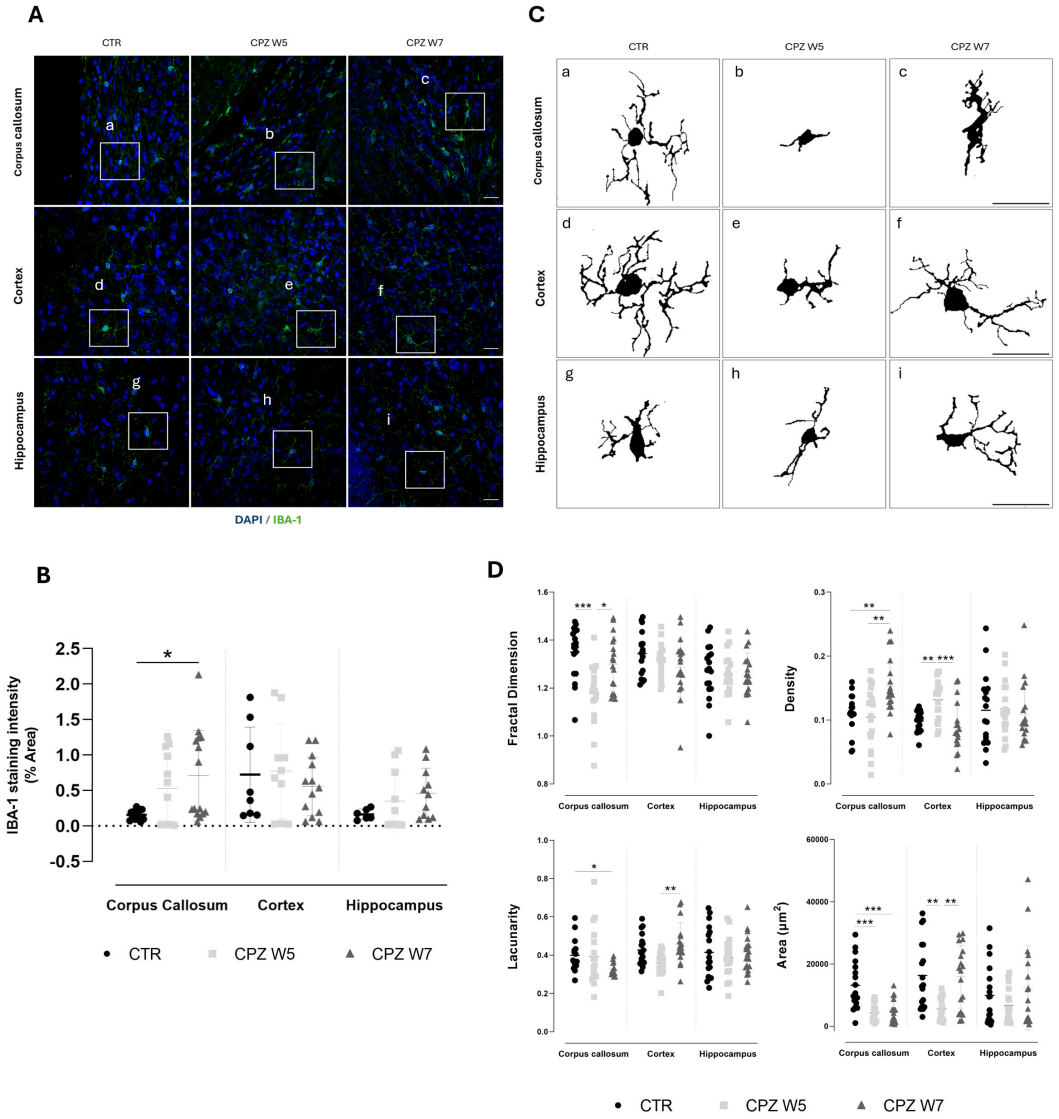
Microgliosis in the brain of cuprizone-intoxicated mice. **(A)** Representative z-stacked photomicrographs of DAPI (blue) and IBA-1 (green) staining in astrocytes from control (CTR) mice, CPZ-intoxicated mice at the demyelination peak (CPZ W5), and CPZ-intoxicated mice during the remyelination phase (CPZ W7) in the corpus callosum (upper panel), cortex layer VI (middle panel),and hippocampal CA1 region (lower panel). Scale bar: 20 μm. **(B)** Quantification of IBA-1-positive area. The region marked by a square in **(A)** is shown at higher magnification in **(C)**, where individual microglia cells outlines were extracted for fractal analysis to assess morphological complexity. **(D)** Summary data and statistical analysis of astrocyte fractal dimension, lacunarity, density, and area. Data is presented as mean ± S.D. of 3 animals per group. Statistical comparisons were performed between all groups. Symbols are absent for non-significant comparisons. *p < 0.05, **p < 0.01, ***p < 0.005.

Analogously, regional myelin alterations are accompanied by changes in microglial density and morphology. Brain cryosections were immunolabeled for IBA-1 - representative images are depicted in **Figure 7A**. In contrast to GFAP, IBA-1 immunostaining revealed greater regional variability (**Figures 7B, C**). A mild increase in IBA-1 signal intensity was observed in the corpus callosum of cuprizone-treated mice, while more subtle elevations occurred in the hippocampus and especially the cortex. Although these differences did not reach statistical significance, they highlight the heterogeneous, region-specific nature of microglial responses, likely influenced by the degree of demyelination and resulting myelin debris, which appeared more pronounced in the corpus callosum and hippocampus when compared to the cortex. Fractal analysis of microglial cells (**Figure 7D**) revealed a significant reduction in both cell complexity (p = 0.0005) and area (p = 0.0005) within the corpus callosum at the peak of demyelination, consistent with a transition to simpler, more rounded morphologies typical of phagocytic microglia. A similar trend was observed in the cortex, where microglia also exhibited reduced cell area (p = 0.0034) and increased density (p = 0.0049), indicative of a phagocytic phenotype. Although not statistically significant (p = 0.0577), the decrease in lacunarity further supports this morphological shift towards activation.

During remyelination, microglia in the corpus callosum showed a recovery in fractal dimension (p = 0.0458), suggesting a re-emergence of ramified morphology. However, sustained reductions in lacunarity (p = 0.0172 versus CTR) and elevated cell density (p = 0.0075 versus CTR) suggest that these cells remained partially active, likely due to the continued presence of myelin debris in this region. In contrast, cortical microglia largely returned to baseline following cuprizone withdrawal, with full recovery of morphological heterogeneity (p = 0.0025), cell area (p = 0.0096), and density (p = 0.0002) - consistent with a reduced burden of demyelination and debris clearance in this area. Hippocampal microglia appeared to follow a similar pattern, albeit less prominently.

## 4 Discussion

### 4.1 Main findings

**Figure 8:**
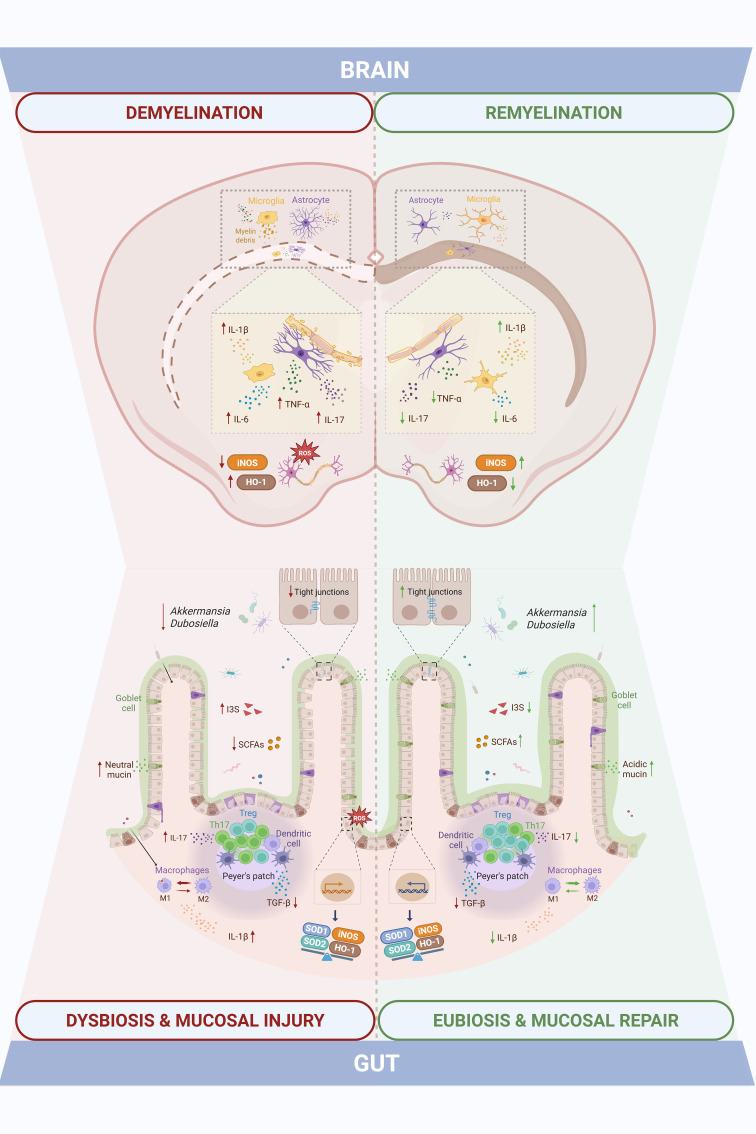
Intestinal mucosal changes paralleling central demyelination and remyelination highlight gut-brain axis dynamics in the cuprizone model of Multiple Sclerosis. Cuprizone-induced demyelination was accompanied by gut dysfunction, including shifts in gut microbiota composition and SCFAs/I3S metabolites, altered mucus and tight junctional integrity, and increased M1 macrophages and Th17 cells with elevated inflammatory (IL-1β, IL-17, TGF-β) and oxidative stress (iNOS, HO-1, SOD1, SOD2) markers. These changes overlapped temporally with central events, as the demyelination peak involved myelin loss, glial activation, neuroinflammation (TNF-α, IL-17, IL-1β, IL-6), and impaired antioxidant defenses (iNOS, HO-1). Both intestinal and central disturbances were partially resolved during the remyelination phase, eliciting a temporal alignment that points to gut-brain axis involvement in the cuprizone model.

To our knowledge, this is the first comprehensive characterization of intestinal mucosal alterations in the CPZ model, a widely accepted preclinical model of reversible CNS demyelination. Our data reveal that CPZ exposure not only triggers well-established central pathology but also induces dynamic, temporally coordinated, and region-specific intestinal responses that closely mirror CNS injury and subsequent repair, as summarized in **Figure 8**.

In the intestinal compartment, cuprizone-intoxicated animals displayed relevant shifts in GM composition and function. Toxin exposure led to a transient depletion of SCFA-producing taxa and enrichment of indoxyl sulfate-producing bacteria. Notably, microbial composition and bacterial metabolite profiles progressively normalized during the remyelination phase, paralleled by an increased abundance of *Akkermansia* and *Dubosiella* genera known to include SCFA-producing strains (42, 43). These observations suggest a recovery of gut microbial homeostasis upon cuprizone withdrawal, that temporally coincides with CNS remyelination. Preclinical and clinical evidence on the beneficial role of *Akkermansia* have been emerging, showing that this bacterial genus establishes a negative correlation with EAE development and MS disability (44, 45).

Despite the absence of overt architectural disruption in the colon, as shown by preserved intestinal layer thickness, CPZ-treated animals exhibited GALT (gut-associated lymphoid tissue) hyperplasia displayed cellular infiltrates and molecular alterations in key barrier-related elements during the intoxication. Perturbations in colonic mucus, a critical component of the gut barrier, were also observed. Goblet cell numbers were transiently reduced, while mucin content per cell increased, likely reflecting a compensatory secretory response. The observed shift toward immature (neutral) mucins during demyelination, followed by a restoration of more mature (acidic) mucins during remyelination, suggests a finely tuned regulation of mucus maturation aimed at re-establishing barrier integrity (42, 46). A similar pattern was observed when assessing tight junctional integrity: while occludin, claudin-5 and ZO-1 gene expression were disrupted at the peak of demyelination, consistent with increased intestinal permeability and compromised barrier function well-documented in both MS patients and EAE models (6, 47), a noteworthy level of recovery took place during remyelination. The increase in ZO-1 protein expression despite reduced mRNA levels suggests post-transcriptional regulatory mechanisms, which warrant further investigation.Immune profiling of gut-associated lymphoid tissue components (Peyer’s patches) further revealed an imbalance in Th17/Treg populations during CPZ exposure, which normalized upon toxin withdrawal. A similar temporal shift was seen in colonic macrophages, which adopted a pro-inflammatory M1 phenotype at week 5, alongside increased expression of IL-17, IL-1β, iNOS and HO-1 and decreased expression of TGF-β, which reverted to an anti-inflammatory M2 phenotype by week 7. Interestingly, macrophage polarization has been shown to significantly impact colon’s homeostasis: M1 macrophages have been demonstrated to reduce goblet cell numbers in colonic crypts and express proinflammatory mediators that damage the intestinal epithelial barrier (e.g.: IL-1β, iNOS), while M2 counterparts foment a pro-regenerative environment (e.g. TGF- β, IL-10), preserving tissue integrity (48, 49). Moreover, intestinal SOD1 expression was reduced during intoxication, likely reflecting CPZ’s copper-chelating activity, given copper’s role as a cofactor for this antioxidant enzyme.

SCFAs are key mediators of intestinal homeostasis, known to promote goblet cell differentiation and mucus production (50, 51), enhance epithelial barrier function through tight junction strengthening (52, 53), and modulate mucosal immune responses by promoting Treg cells and suppressing Th17 polarization (54). Several clinical studies have reported lower SCFAs concentrations in the feces and serum of MS patients (55–57). In particular, levels of serum SCFAs seem to associate with changes in circulating immune cells and biomarkers involved in MS development (56), as well as with disability and lesion burden (55). Moreover, supplementation with propionate has proven to ameliorate the disease course and increase functional Treg cells numbers while also decreasing Th1 and Th17 subsets (17) and butyrate has shown to promote remyelination *in vitro* (29). The dynamic fluctuation of SCFAs levels across the intoxication and recovery phases may suggest their potential contribution to the temporal coordination of epithelial barrier restoration, innate and adaptive immunomodulation and CNS repair. Of particular interest is the expansion of *Dubosiella newyorkensis* during remyelination, previously linked to rebalancing the Th17/Treg axis and redirecting tryptophan metabolism toward the kynurenine pathway, thereby reducing the production of neurotoxic indoxyl sulfate (43). These findings may suggest a potential role for *D. newyorkensis* as a potential microbial contributor to gut, and possibly, CNS recovery following demyelination. Given the known inter-individual variability in intestinal microbiota and mucosal immunity and the small sample sizes employed in this study, our results require validation in larger cohorts or independent replicates. Nevertheless, the consistency of trends across multiple readouts supports the robustness of our main conclusions.

In the CNS, hallmark features of CPZ-induced pathology were confirmed, including region-specific demyelination. The most affected areas were the corpus callosum and hippocampus, where myelin loss and glial activation were prominent. This distribution aligns with prior reports showing that cortical demyelination is less severe and occurs with delayed kinetics (58, 59). The cortex, however, demonstrated a more efficient remyelination response, consistent with observations in both MS patients and cuprizone models (60–62). While MBP expression suggests structural recovery, further studies are needed to determine whether remyelination also restores axonal function.

As expected, the expression of classical pro-inflammatory cytokines (TNF-α, IL-6, IL-1β, IL-17) peaked during demyelination in whole brain homogenates, while most markers declined during remyelination, TNF-α and IL-17 remained elevated and IL-1β expression increased further. This cytokine plays a dual role in MS and cuprizone models (63, 64). Although often associated with neurotoxicity, IL-1β is also required for efficient remyelination via promotion of OPC differentiation through insulin growth factor 1 (IGF-1) signaling (65). These results support the concept that tightly regulated, non-resolving inflammation may be necessary for optimal remyelination. In line with previous findings, oxidative stress responses were modest. While HO-1 expression was upregulated, consistent with activation of the Nrf2 pathways, SOD1 and SOD2 levels remained largely unchanged, possibly due to their lower inducibility (66, 67).

Glial responses mirrored the regional vulnerability seen in myelin dynamics, indicating distinct susceptibility to CPZ-induced CNS damage. Astrocytes remained reactive and disorganized in the corpus callosum and hippocampus even after toxin withdrawal, whereas cortical astrocytes returned to a more quiescent state. This suggests region-dependent differences in glial reactivity and debris clearance towards repair efficiency. While sustained astrocyte activation is often seen as detrimental, recent evidence highlights their reparative roles, including HO-1 production, metabolic support to OPCs, and recruitment of phagocytes for myelin debris clearance (68, 69). Microglial activation, as assessed by IBA-1 staining and fractal analysis, was particularly evident in the corpus callosum, displaying a shift toward a rounded, phagocytic morphology during demyelination (70). In contrast, cortical microglia showed only subtle changes in IBA-1 staining intensity, consistent with limited demyelination, but fractal analysis revealed morphological changes consistent with phagocytic activity and consequent myelin debris clearance. In the hippocampus, intermediate microglial activation paralleled the degree of myelin loss observed.

### 4.2 Conclusion and future perspectives

In summary, this study provides the first integrative temporal characterization of gut–brain axis components in the CPZ model of reversible demyelination. By evaluating GM composition and function, intestinal barrier integrity, mucosal immune responses, and inflammation in parallel with CNS lesion dynamics across both demyelination and remyelination phases, we demonstrate that intestinal dysfunction closely mirrors central pathology. These findings, obtained in the absence of peripheral autoimmunity, suggest that gut alterations are not merely secondary to systemic immune activation, but may constitute an integral component of the demyelination–remyelination continuum, recapitulated by the CPZ intoxication. Although CPZ primarily targets oligodendrocytes, the coordinated intestinal responses observed point to broader systemic effects that may influence glial activity and neuroinflammation through circulating microbial metabolites or immune mediators. Notably, this is the first report to identify a robust intestinal immune component in the CPZ model, expanding beyond microbiota profiling to include detailed structural, barrier-related, and immunological analyses of the intestinal mucosa. By synchronously analyzing central and intestinal parameters, this study offers a systems-level perspective on gut–brain interplay during both injury and repair phases, contributing new insights into the temporal coordination of these compartments in this preclinical model of MS.

Several limitations must be also acknowledged. While associations were found between microbiota composition, microbial metabolites, and CNS responses, causality cannot be established from the current dataset. Although evidence on CPZ’s direct effects on the gut is scarce, the possibility that CPZ independently influences both the intestinal and central compartments cannot be excluded. Therefore, it is not conceivable to infer a direct interdependent relationship between them yet. Mechanistic studies using germ-free or antibiotic-treated mice, fecal microbiota transplantation, or targeted metabolite interventions will be essential. Additionally, future work should explore OPCs dynamics, immune cell infiltration and blood–brain barrier integrity to complement the current findings. While fractal analysis of glial morphology points to phagocytic activation, myelin debris clearance efficiency was not directly assessed. Hence, subsequent analyses underscoring the observed regional de/remyelination and glial reactivity disparities are warranted. Still, this work establishes a methodological framework for investigating gut–brain interactions in experimental MS and lays the groundwork for future preclinical testing of gut-targeted interventions (e.g., pre-, pro-, syn-, and postbiotics) to enhance remyelination and promote neurorepair.

## Data Availability

All relevant data is contained within the article: The original contributions presented in the study are included in the article/supplementary material. Further inquiries can be directed to the corresponding author.
