# Supplementary material for: Intestinal mucosal alterations parallel central demyelination and remyelination: insights into the gut-brain axis in the cuprizone model of multiple sclerosis
Source: Front Immunol. 2025 Oct 28;16:1682183. doi: 10.3389/fimmu.2025.1682183 (PMC12602228; doi:10.3389/fimmu.2025.1682183)

Supplementary Material

Supplementary Table 1. Body weight change (Δ weight) and relative organ weights of gut, brain, liver, and thymus. Body weight variation (Δ weight) is presented from baseline (week 0) until sacrifice and organ weights normalized to total body weight expressed as milligrams per gram of body weight (mg/g body weight) from controls (CTR), CPZ-intoxicated mice at the demyelination peak (CPZ W5), and CPZ-intoxicated mice during the remyelination phase (CPZ W7). Data is presented as mean ± SD of 8 animals per group. Statistical comparisons were performed between all groups. Symbols are absent for non-significant comparisons. *p < 0.05 versus CTR; **** p < 0.0001 versus CTR; ++ p < 0.01 versus CPZ W5; ++++ p < 0.0001 versus CPZ W5.

	CTR	CPZ W5	CPZ W7
**Δ Weight (g)**	3.300 ± 0.912	0.400 ± 0.809****	3.388 ± 1.204++++
**Relative organ weight (mg/g body weight)**	**CTR**	**CPZ W5**	**CPZ W7**
**Gut**	7.144 ± 0.454	8.038 ± 0.602*	8.025 ± 0.624*
**Liver**	7.049 ± 0.309	8.563± 1.004*	7.338 ± 1.775
**Brain**	3.303 ± 0.690	3.593 ± 0.585	3.460 ± 0.437
**Thymus**	1.987 ± 0.511	1.736 ± 0.198	2.629 ± 0.572++

Supplementary Table 2. Overview of the fluorochrome-labeled monoclonal antibodies used for multiparametric flow cytometry analysis.

Antibody	Conjugate	Clone	Brand	Cat#
CD3	FITC	145-2C11	BD Pharmingen™	553062
CD25	PE	PC61	BD Pharmingen™	553866
CD4	PE-Cy7	RM4-5	BD Pharmingen™	561099
FoxP3	AF647	MF23	BD Pharmingen™	560401
CD45	APC-Cy7	30-F11	BD Pharmingen™	557659
RORγt	BV421	Q31-378	BD Horizon™	562894
IL-17A	BV510	TC11-18H10	BD Horizon™	564168

Supplementary Figure 1. Representative gating strategy applied to cell suspensions from Peyer's patches for the identification of Th17 and Treg cells. Doublets were first excluded, followed by the selection of $CD45^{+}$ leukocytes. $CD3^{+}CD4^{+}$ T cells were then gated, within which Th17 cells ($CD3^{+}CD4^{+}ROR\gamma t^{+}IL\text{-}17A^{+}$) and Treg cells ($CD3^{+}CD4^{+}FoxP3^{+}CD25^{+}$) were identified.

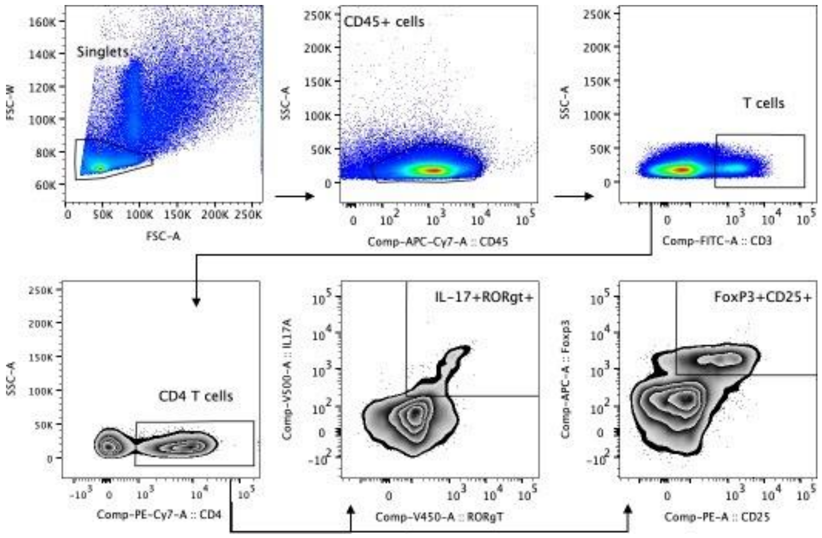

Supplementary Figure 2. Fractal analysis of cell morphology in GFAP-stained tissue. Tissue images (a) were transformed into binary images (i) using a defined threshold, from which single cells were randomly selected (j). Each cell image was edited to clear the background and fill gaps in the branches of the selected cell, obtaining a filled shape (k) and its pairwise outline (l), which was used for fractal analysis. Scale bar = 20 µm.

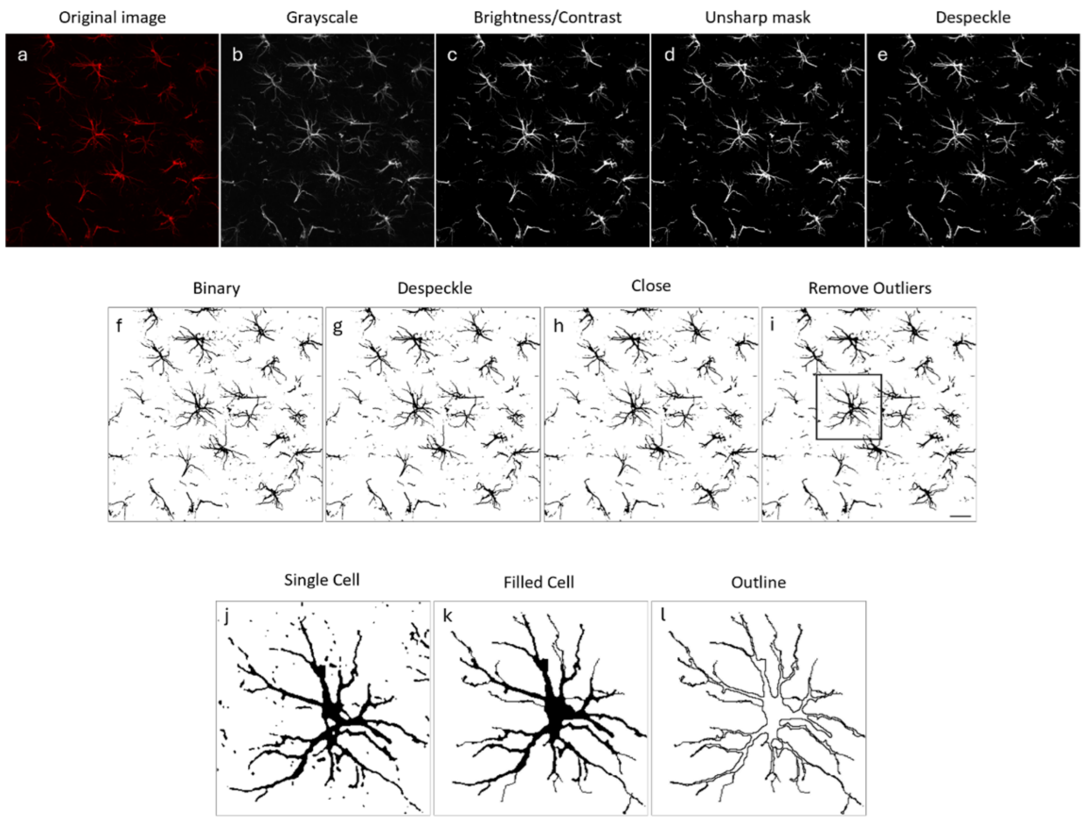

Supplementary Figure 3. Summary of the count of total (CD68⁺), M1 (CD86⁺) and M2 (CD206⁺) macrophages in the colon of CPZ-intoxicated mice. Data is presented as mean ± S.D of 4 animals per group. Statistical comparisons were performed between all groups. Symbols are absent for non-significant comparisons. ** $p<0.01$; *** $p<0.005$; **** $p<0.001$.

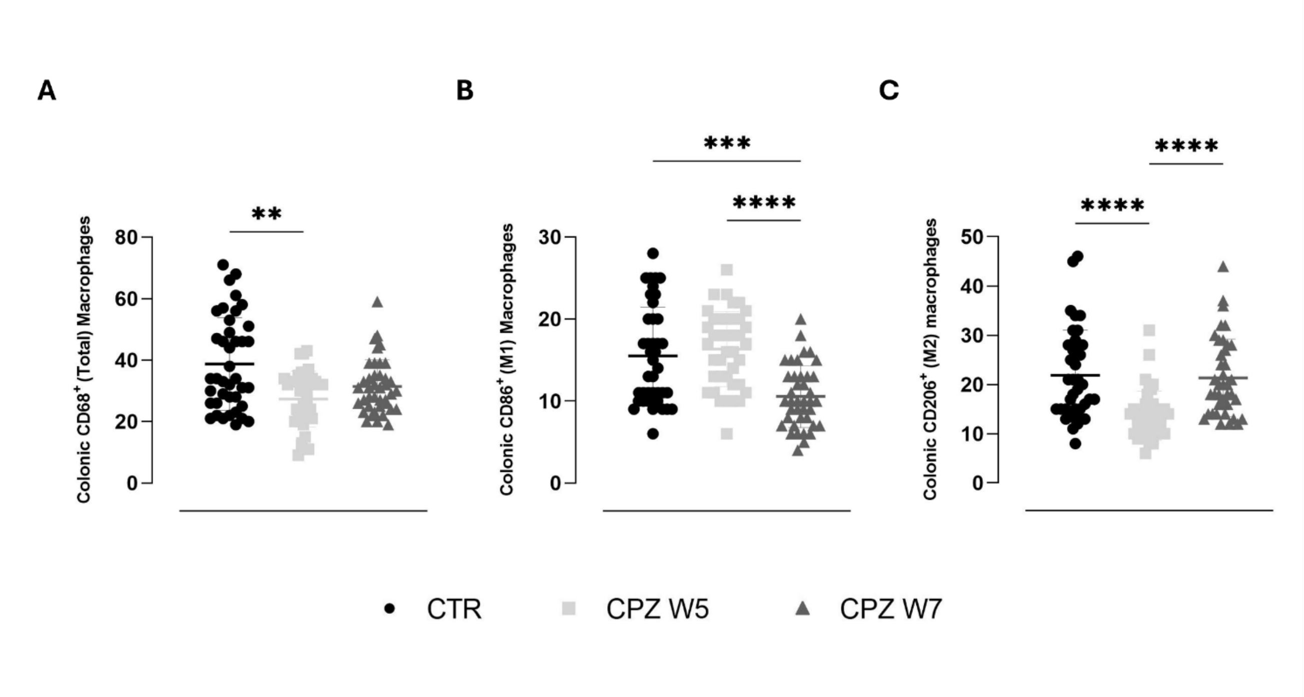

Supplement: Supplementary file 1 [file SupplementaryFile1.pdf]
